# Conservative bias, selective political exposure and truly false consensus beliefs in political communication about the ‘refugee crisis’ in Germany

**DOI:** 10.1371/journal.pone.0259445

**Published:** 2021-11-04

**Authors:** Dominic Burghartswieser, Tobias Rothmund

**Affiliations:** 1 University of Koblenz-Landau, Mainz, Germany; 2 Friedrich-Schiller University Jena, Jena, Germany; University of Connecticut, UNITED STATES

## Abstract

The rise of digital media has increased the opportunities for individuals to self-select political content online. This development has stimulated empirical research on how people select political information, especially when political beliefs are at stake. In the present paper, we tested a series of theory-derived assumptions about antecedents and consequences of selective exposure to confirmative political information and opinions in the digital arena. We conducted an online survey with German Internet users (N = 897, April 2016) and assessed political attitudes, media use and general beliefs in the context of the so-called “migration crisis”. 28% of the participants in our sample reported exposure to a confirmative information environment. They are more likely to hear or read about political opinions on migration and political asylum that are similar to their own compared to cross-cutting content. We found no evidence for the assumption that the technological affordances of the Internet foster this form of selective political exposure. Instead, our analyses indicate that conservatism is a positive predictor of selecting confirmative information environments when it comes to migration and political asylum. We also gathered evidence that this relation is mediated by perceived threat and that selective political exposure is linked to truly false consensus beliefs. Our findings inform supply- and demand-side explanations of selective political exposure online. We discuss the relevance for psychological theories about the motivational underpinnings of selective exposure.

## Introduction

The rise and proliferation of digital media in political communication is accompanied by public and scientific debates about their potential (psychological) consequences [1]. One debate is concerned with the question of whether and how digital media consumption may foster a more balanced or rather a more one-sided perspective on social issues in political debates. On the one hand, the Internet or, more specifically, social networking sites are supposed to provide exposure to political differences and cross-cutting perspectives [2, 3]. On the other hand, authors like Sunstein [4] argued that the Internet is a high-choice media environment [5] which favors the emergence of so-called echo chambers, in which individuals exchange their political positions primarily with like-minded others. Bennett and Iyengar articulated similar concerns when they predicted a „fragmented audience in an era of selective exposure”[1].

The metaphor of an “echo chamber” is frequently used to describe a situation in which individuals are primarily exposed to political opinions and attitudes of like-minded others. However, the terminology entails some problems. First, the term lacks conceptual clarity. There is no consensus on how to conceptualize an echo chamber situation. This is especially problematic for measurement. Many studies assess communication only in a single medium to operationalize exposure to political opinions [6–10]. However, individuals typically use more than one medium to seek political information and the one-sided use of one medium could be compensated by the use of a second [11]. Such conceptual ambiguities make it difficult to compare studies and present an obstacle for cumulative research. Second, the term echo chamber conveys a spatial notion of what is, in reality, a communicative practice. People do not enter an echo chamber like a room, but select communication partners or exchange information in ways that result in *selective political exposure*. This kind of exposure to specific political arguments and beliefs is characterized by a significant imbalance between pro- and counter-attitudinal content where the former prevails [10].

In the present paper, we aim to investigate potential antecedents and consequences of selective political exposure. We derived hypotheses about the boundary conditions of this phenomenon from three different theoretical accounts. These accounts can broadly be categorized as supply-side and demand-side explanations. Supply-side explanations focus on characteristics of the medium or a channel itself. We investigated the assumption that characteristics of the Internet make exposure to like-minded political beliefs and attitudes generally more likely compared to the use of other information sources (*technological hypothesis*) [12]. Demand-side explanations focus on the psychological preconditions of media recipients. The common assumption of these accounts is that specific psychological characteristics make some individuals more likely to select confirmative information environments compared to others. We investigate two conflicting hypotheses that postulate symmetric and asymmetric relations with political ideology. Symmetric relations are in line with a series of theoretical accounts such as cognitive dissonance theory [13], uncertainty-identity theory [14] and identity-based models of political ideology [15]. Based on these accounts, people with stronger political preferences should be more likely to report selective political exposure (*ideological strength hypothesis*). This relation should be symmetrical for liberals and conservatives. Asymmetrical effects can be expected based on the motivated social cognition framework [16] which posits that conservatives are generally less open to dissenting political information. In line with this theoretical account, we expect a positive relation between selective political exposure and political conservatism (*conservatism hypothesis*).

We also aim to investigate potential psychological effects of selective political exposure. More precisely, we focus on the *truly false consensus effect* [17] and test whether exposure to confirmative information environments is related to the likelihood of overestimating the share of people with similar political attitudes and beliefs.

### Selective political exposure in high-choice information environments

The spread of digital media has significantly changed the relationship between audiences and media, that is, the relationship between media demand and supply. In the heyday of traditional mass media, a handful of news channels were able to cater almost an entire national news audience [18]. Professional norms led to a very similar set of news shared by these few channels and chances were high that a certain amount of common political knowledge would develop as a byproduct of regular media consumption [1]. With the proliferation of cable television and digital media, technological innovations resulted in more control and lower costs for users in terms of both content creation and selection [19]. Subsequently, and in order to cope with an abundant media supply, demand side information selection processes gained growing importance [1]. In communications research, these changes are reflected in the term *high-choice media environment* [5].

As a result of these shifts, the concept of *selective exposure* received renewed attention. Introduced by Lazarsfeld, Berelson and Gaudet [20], it describes the phenomenon that individuals tend to approach confirmative information, avoid dissenting information, or, sometimes, both [21]. The transition to a high-choice media environment where “news media options are endless” [22], greatly enhanced preconditions for selectivity. In the Internet, this trend is enhanced by an increasing competition for the attention of audiences among media suppliers, a competition that intensifies the “tendency for media to cater to audience demands” [5]. Several news media began to follow a ‘”niche news” paradigm’ [1], producing content adjusted to specific informational demands [22]. Unlike traditional mass media news programs with large and diverse audiences, recent trends thus seem to promote „targeted channels for targeted audiences”[23]. As part of the distribution of news content, algorithms obey the same logic. Using prior consumption patterns as starting point in the attempt to identify and meet specific audience demands, algorithms, too, tend to limit the diversity of news individuals encounter [24]. These characteristics of news production and distribution give support to the assumption that the use of high-choice information environments like the Internet will increase selective political exposure.

Empirical research on this assumption is mixed. On the one hand, several studies gathered evidence for *selective political exposure* in online environments [3, 8, 10, 25–27]. On the other hand, evidence for an increase in selective political exposure in high compared to low-choice media environments is still limited. A major methodological problem in this line of research is that studies typically investigate only one source or medium of information and, thus are not able to compare across types of media [11]. There are a few exceptions. Stroud [28], for instance, investigated partisan selective exposure across different types of media and found evidence for its existence in newspapers, talk radio, cable news and the Internet. However, the effect was weak for users of newspapers and most pronounced in the case of Internet users. Similarly, Garrett [21] reported that using the Internet, as opposed to offline media, was linked to a higher likelihood of selecting opinion-reinforcing information. To the contrary, Barnidge [29] reported that the use of social networking sites was related to more frequent exposure to political dissent compared to face-to-face communication.

Our first hypothesis aims at addressing this gap in comparative research. We compare four sources of information, the Internet, TV, Newspapers, and face-to-face communication. In terms of opportunities for choice, we expect the Internet to provide the greatest amount of selectivity. With regard to newspapers, we think of more or less inalterable compilations of pre-selected information, whereas we expect television, with its ‘flow’ of information and the opportunity to switch between channels to fall in between the Internet and newspapers in terms of its potential for selectivity. We have no particular hypothesis for face-to-face communication. Since these predictions are based on the assessments of technological affordances, we called the first hypothesis *technological hypothesis*.

**H1**. The use of high-choice media environments, especially the Internet, predicts selective political exposure.

### Psychological accounts for selective exposure in political communication

A disputed question in research on selective political exposure regards the role of ideology and partisanship [8, 30]. There is strong evidence that the psychological affiliation with political ideas or parties fosters selective political exposure [22]. However, the nature of this relation is still inconclusive. A central question here is whether this relation is symmetrical or asymmetrical [6, 9, 26, 28].

There are different theoretical reasons to expect symmetric patterns of selective political exposure. Cognitive dissonance theory [13] is the most commonly used theory to explain selective exposure [12, 31]. Since cognitive dissonance is supposed to be as general in nature as hunger or thirst [32], it should motivate liberal and conservative individuals in the same manner. Hence, without further auxiliary assumptions, cognitive dissonance theory speaks in favor of the expectation that liberals are as likely as conservatives to select politically confirmative information environments in order to avert cognitive dissonance. Similar predictions can be derived [8] from uncertainty-identity theory [14]. The theory is concerned with group identity processes under conditions of uncertainty and threat. One if its core assumptions is that individuals are motivated to “effectively reduce uncertainty” [14] and that group identification is a useful means to this end. Group identification serves this function particularly well, when people can identify with “a clearly structured high-entitativity group with sharp boundaries, unambiguous membership criteria, highly shared goals, and consensus on group attributes” [14]. Among the groups that are supposed to match these requirements are radical political groups, left and right. In line with dissonance theory and uncertainty-identity theory, we thus hypothesize that individuals with stronger ideological belief systems (both liberal and conservative) are more strongly motivated to reduce uncertainty and, thus, more susceptible to selective political exposure. We labelled the second hypothesis *ideological strength hypothesis*.

**H2**. The strength of ideological beliefs, left and right, is a positive predictor of selective political exposure.

There is some evidence that the relation between ideological beliefs and selective political exposure is not symmetrical but asymmetrical [7, 33–36]. Most studies draw on motivated social cognition [16] as a theoretical framework [8, 36]. The basic assumption in this line of thinking is that liberals and conservatives differ in their need to manage uncertainty and threat. More specifically, conservatives are less willing to tolerate ambiguity and more motivated to avoid uncertainty compared to liberals. Motivated social cognition is part of a tradition that dates back to the idea of an authoritarian personality [37]. Empirically, it is supported by consistent differences between conservatives and liberals in terms of psychological dispositions such as dogmatism, cognitive and perceptual rigidity, personal needs for order and structure, need for cognitive closure, intolerance of ambiguity or (in)tolerance of uncertainty [38]. These motives are supposed to interfere with information selection processes and to promote selective exposure [16]. Based on this theoretical account, we hypothesize that conservatives will show stronger patterns of selective political exposure. For evident reasons, we labelled the third hypothesis *conservatism hypothesis*.

**H3**. Political conservatism is a positive predictor of selective political exposure.

The *ideological strength hypothesis* and the *conservatism hypothesis* share some common theoretical ground. In both cases, avoidance of uncertainty and threat is conceived as the underlying motivational drive that links political ideology with selective political exposure. Prior studies support this assumption. Fischer and colleagues [39] in a series of five studies found that heightened perceptions of threat, if relevant in the context of decision making, lead to more selective exposure. Lavine, Lodge and Freitas [40] investigated the effect of perceived threat on high- and low-authoritarians and found that it lead to more selective exposure in case of the former, but not the latter. We aim to directly test this assumption in our study by investigating whether perceived threat mediates the relation between (a) ideological strength and selective political exposure, or (b) political conservatism and selective political exposure.

### Psychological effects of selective political exposure in political communication

The societal consequences of selective political exposure on democratic deliberation are a matter of concern [41]. Exposure to disagreement is key in democratic deliberation where different kinds of interests are held and weighed against each other [42]. Accordingly, it is hypothesized that exposing citizens to different viewpoints improves their ability and motivation to participate in democratic processes [43]. Empirical investigations are rare, but there is some support for this assumption. Exposure to disagreement enhanced people’s ability to generate arguments, in particular concerning the reasons why others might disagree with their views [42]. In addition, exposure to disagreement increased political tolerance and democratic legitimacy, that is, the legitimacy individuals concede to cross-cutting opinions in political debates [44]. On the other hand, individuals exposed to larger amounts of political dissent may also, by way of either political ambiguity or social accountability, be discouraged to participate in politics [41].

Wojcieszak and Price [45] investigated a cognitive effect of exposure to political disagreement. They gathered evidence that encountering political disagreement reduced the *false consensus effect* [46], a tendency of individuals to overestimate the amount of people with attitudes and behavioral choices similar to their own. Wojcieszak and Price replicated this effect with regard to participants’ perceived consensus on three contentious socio-political issues. Exposure to disagreement attenuated the false consensus effect and appeared “to mitigate the tendency for people to attribute their views to the general population” [45].

In the present paper, we aim to complement the work of Wojcieszak and Price [45] by testing whether selective political exposure is linked to a higher false consensus effect compared to other, more balanced, informational diets. Contrary to the authors, we use a measurement technique that allows to assess the accuracy of individual’s perceptions of similarity. This technique is associated with the notion of a *truly false consensus effect* and was developed in response to criticism of the original concept [17]. The false consensus effect postulated a “false” perception of consensus and an egocentrically biased projection, respectively, if individuals who endorsed an attitude or behavior also thought of the latter as more common in a certain population [46]. However, some attitudes or behavior are more common than others and estimates that match this fact can hardly be called “false”; in addition, it is completely valid, in terms of inductive reasoning, to use one’s own position as a piece of evidence in an effort to estimate the characteristics of a certain population [47]. Consequently, Krueger and Zeiger developed a measure that correlated item endorsements, not with individuals’ estimates, but with “the differences between estimated and actual consensus” [17], thus indicating a *truly* false consensus effect.

The supposed underlying mechanisms were not affected by the critique. Selective exposure has always been considered a possible source of the false consensus effect [46]. More specifically, selective exposure to a biased sample of people or ideas will likely influence an individual’s ability to recall earlier instances of (dissenting) attitudes or behavior [45]. Likewise, Bauman and Geher [48] found that exposure to both sides of contentious social issues lead to less biased estimations of consensus. Based on this rationale, we test the assumption that a truly false consensus effect is negatively associated with the relative amount of exposure to disagreement. We thus expect selective political exposure to be associated with a stronger truly false consensus effect compared to more balanced patterns of exposure to political information.

**H4**. Selective political exposure is a positive predictor of the truly false consensus effect.

### The present research

We tested our hypotheses in the context of a political debate on migration and asylum in Germany in 2016. Mostly as a result of the Syrian civil war that broke out in 2011 as well as due to the activities of the self-proclaimed *Islamic State* in both Syria and Iraq, millions of refugees had to leave these countries. While most of the refugees settled in neighboring countries, a significant amount headed towards Europe. Their numbers peaked in the years 2015 and 2016 with 476,510 and 745,155 applications for asylum in Germany, respectively [49]. In this context, Federal Chancellor Angela Merkel in 2015 announced that Germany will keep its borders open for asylum seekers, a decision that led to intense public controversy. We believe that this political topic is suited to investigate the antecedents and consequences of confirmative information environments. At the time of data collection in spring 2016, the so-called refugee or migration crisis was of extraordinary public relevance. The topic was discussed in a controversial manner among liberals and conservatives, especially in social media. This made it likely that most respondents had encountered information in different forms of media outlets and even developed a personal opinion.

## Method

### Sample

We used the survey website SoSciSurvey (www.soscisurvey.de) to assemble an online questionnaire. Data collection was conducted between March 30 and April 17, 2016 by keyfacts (www.keyfacts-gmbh.de) which recruited a sample of German adults. 1,273 participants started the survey, 993 finished it (attrition rate 22%). Age, gender and educational level were quoted during the online recruiting. People who did not match the quota were excluded at the beginning of the study and were not able to proceed in the questionnaire. Participants who finished the survey faster than 50% of the Median (N = 96) were excluded. The final sample included N = 897 participants (50.12% women). The age distribution ranged from 13 years to 87 years (M = 48.86, SD = 17.02). Education was almost equally distributed with regard to low (“Volks- oder Hauptschulabschluss”, 39.7%), middle (“Real- oder Oberschulabschluss”, 29.7%), and high (“Fachhochschule oder Hochschulreife”, 28.4%). 1.1% were still seeking for a higher level of education and another 1.1% of the participants had no educational degree. 42.6% of the participants were “not employed”, 14.8% “half-time employment”, 36.9% “full-time employment” and 5.8% “pupil or student”.

### Measures and procedure

The measures are described in the order they’ve been presented in the questionnaire. If not noted otherwise, response scales ranged from 1 (completely disagree) to 6 (completely agree). The dataset, the R-script and the complete survey (including items that we did not use in the present study) are accessible via https://osf.io/mpqgb/.

#### Demographics

We assessed gender, age, highest level of formal education and employment status. Distributions of gender, highest level of formal education and employment status in our sample are similar to the distributions in German society [50]. It is important to note that “not employed” also includes pensioners. Mean age in our sample (48.9 years) was above the population average in the year of data collection (44.3 years) [51].

#### Attitude towards political asylum

We assessed attitudes towards political asylum with eight items measuring normative beliefs (e.g., “Asylum seekers without certification of nationality should be rejected in a fast process”) and eight items measuring factual beliefs (e.g., “Asylum seekers take away jobs from the native population”) about refugees and political asylum. The 16 items were combined into a single attitude measure (Cronbach’s α = .89).

#### Truly false consensus beliefs about public opinion towards political asylum

**Fig 1:**
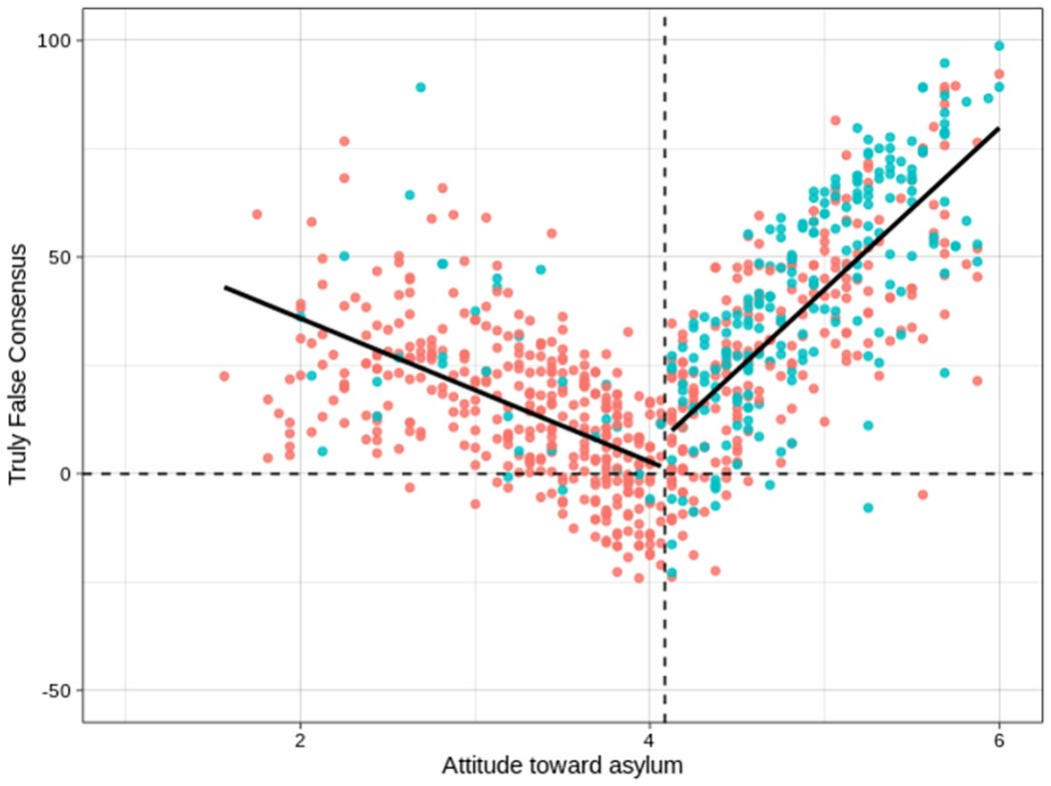
Truly false consensus scores by attitudes towards political asylum.

Participants were asked to estimate the distribution of attitudes towards political asylum in the general public. We used two items (“Please estimate the share of people in the German society who are more critical towards political asylum compared to you?”; “Please estimate the share of people in the German society who are more open towards political asylum compared to you?”). The two items ranged from 0% to 100% and were adapted from Rattinger, Roßteutscher, Schmitt-Beck and Weßels [52]. We inverted the second item and calculated a mean score of both items indicating participants’ estimation of the share of people in the German society with more critical attitudes towards political asylum. By locating the rest of society relative to themselves, participants implicitly provided a percentage estimation of their own position in society with regard to attitudes towards political asylum. Based on our attitude measure (see above), we also calculated percentiles for each individual indicating the actual position in our sample with regard to his / her attitude towards political asylum. Eventually, we thus have two items, the first one indicating a person’s estimate of the share of people in German society with attitudes more negative towards asylum, the second one indicating the actual share of people in our sample with attitudes more negative towards asylum. The latter score was subtracted from the estimate, the result thus indicating the difference between estimate and actual position in our sample (difference score). A last step was necessary to bring the over- or underestimation in relation to the participant’s own positive or negative attitude towards political asylum. To this end, participants were divided into two groups with positive and negative attitudes towards political asylum, respectively, using the mean attitude score (M = 4.08, SD = 0.96) as threshold. If a person held a *negative* attitude towards political asylum (above the sample mean), the difference score served as an estimate of truly false consensus, since a positive result would indicate the overestimation of the proportion of the participant’s own (“more critical”) group which was perceived as closer to, and thus more representative of, the center of the distribution than it actually was. If a person held a *positive* attitude towards political asylum (below the sample mean), the result of the difference score had to be inverted, since in this case a negative result would indicate the overestimation of the own (“more open”) position which was perceived as closer to, and thus more representative of, the center of the distribution than it actually was. Hence, estimations indicated false consensus if, relative to the actual position, they tended towards the center of the distribution (50^th^ percentile), reflecting the assumption that those political views are perceived as more consensual and prototypical for society, respectively, that are closer to the political center. This rationale is supported by the fact that the distribution of attitude scores resembles a normal distribution, albeit slightly skewed to the right. For both groups more positive values on our final false consensus score indicated a stronger truly false consensus effect, the numbers signifying the difference in percentage points between estimate and actual position in our sample (see Fig 1).

Truly false consensus scores on the y-axis by attitude towards political asylum on the x-axis. Blue dots indicate confirmative information environments, while red dots indicate other (disconfirmative, mixed, neutral) information environments. The mean attitude score (M = 4.08, SD = 0.96) was used as threshold to assign individuals to positive and negative attitudes, respectively, and is marked by the vertical dashed line. The horizontal dashed line corresponds to a truly false consensus score of zero. Continuous lines indicate linear regression lines.

#### Perceived threat

Participants rated six items (e.g., “I fear negative consequences for our society as a result of the refugee crisis.”) about the influence of the migration crisis on German society. The items were adapted from Gollwitzer and Keller [53]. We dismissed the last two items which were not politically neutral and combined the remaining four items into a scale (Cronbach’s α = .82).

#### Sources of information about the migration crisis

Participants estimated how often they used TV, Newspaper, Internet and face-to-face conversations as a source of information about the migration crisis (e.g., “How often do you inform yourself about the migration crisis on TV?”). The four items were adapted from Baumgartner and Morris [54] and used a 6-point response scale (1 ‘never’, 2 ‘every other month’, 3 ‘every other week’, 4 ‘weekly’, 5 ‘a couple of times a week’ and 6 ‘daily’).

#### Information environments

We used an indirect measure for selective political exposure to information about the migration crisis by slightly modifying a procedure from Vaccari et al. [10]. Participants responded to two items (“How often do you hear/read statements of people strongly argueing against the admission of refugees?”; “How often do you hear/read statements of people strongly argueing in favor of the admission of refugees?”) using a 6-point response scale (1 ‘never’, 2 ‘every other month’, 3 ‘every other week’, 4 ‘weekly’, 5 ‘a couple of times a week’ and 6 ‘daily’). We dichotomized the measures of exposure to pro- and anti-refugee statements into ‘rarely’ (for ‘never’, ‘every other month’ or ‘every other week’) and ‘frequently’ (for ‘weekly’, ‘a couple of times a week’ and ‘daily’) on the basis that exposure should at least be part of an individual’s weekly routine to be classified as ‘frequently’. Based on this 2x2 matrix, we assigned participants to one of four kinds of information environments: neutral, mixed, confirmative and disconfirmative. If participants’ exposure to pro- and con-refugee communication content was rare, they were assigned to the *neutral information environment* about migration politics. If exposure to pro- and con-refugee communication content was frequent, they were assigned to a *mixed information environment* about migration politics. Assignment to the other two information environments depended on the participant’s personal attitude towards political asylum. The sample mean of our attitude towards political asylum measure (M = 4.08, SD = 0.96) was used to split participants into two groups with positive and negative attitudes towards political asylum, respectively. If participants were frequently exposed to con-refugee statements, but rarely to pro-refugee statements, while holding a negative attitude towards political asylum themselves, they were assigned to a *confirmative information environment* about migration politics. Analogously, if participants were frequently exposed to pro-refugee statements, but rarely to con-refugee statements, while holding a positive attitude towards political asylum themselves, they were also assigned to a *confirmative information environment* about migration politics. Finally, if participants were frequently exposed to pro-refugee statements, but rarely to con-refugee statements, while holding a negative attitude towards political asylum themselves, they were assigned to a *disconfirmative information environment* about migration politics. Likewise, if participants were frequently exposed to con-refugee statements, but rarely to pro-refugee statements, while holding a positive attitude towards political asylum themselves, they were also assigned to a *disconfirmative information environment* about migration politics. The frequencies of the information environments are displayed in Table 1. Mixed information environments were most common in our sample (43.8%), followed by confirmative information environments (27.6%), neutral information environments (15.9%) and disconfirmative information environments (12.7%).

**Table 1:** Self-reported information environments.

	Mixed	Neutral	Confirmative	Disconfirmative	Total
Left	98 (49.7%)	28 (14.2%)	37 (18.8%)	34 (17.3%)	197
Center	235 (42.9%)	98 (17.9%)	147 (26.8%)	68 (12.4%)	548
Right	49 (38.6%)	12 (9.4%)	58 (45.7%)	8 (6.3%)	127
NA	2 (50.0%)	1 (25.0%)	-	1 (25.0%)	4
Total	384 (43.8%)	139 (15.9%)	242 (27.6%)	111 (12.7%)	876

Note. Frequency of self-reported information environments across aggregated political attitudes: ‘Left’ (‘Ideological self-placement’: 1–4); ‘Center’ (5–7); ‘Right’ (8–11). Four instances could not be attributed because of missing scores. Percentages are calculated horizontally. In 21 instances (N = 897) we could not assign participants to one of the four information environments.

Finally, we used one-item measures to assess *political interest* (“In general, how strongly are you interested in politics?”) and *ideological self-placement* (“Concerning political attitudes in general, people often refer to ‘left’ and ‘right’. Where on this dimension would you locate yourself politically?”). Participants were asked to indicate their political interest on a response scale ranging from 1 ‘not at all’ to 6 ‘very strongly’ and ideological self-placement on a response scale ranging from 1 ‘extreme left’ to 11 ‘extreme right’.

## Results

In a first set of analyses, we tested our hypotheses about potential predictors of participants’ exposure to a confirmative information environment about migration politics (selective political exposure). In a second part of the results section, we investigated the link with truly false consensus, a potential psychological consequence of selective political exposure. Bivariate correlations of our main variables are depicted in Table 2, means of main variables separated by information environments are depicted in Table 3. Since multiple hypotheses were tested using the same data, we adjusted the p-value indicating significance to .01 to account for an increased likelihood of rare events.

**Table 2:** Intercorrelations between main variables.

	2.	3.	4.	5.	6.	7.	8.	9.	10.	11.	12.
1. Age	-.01	.26***	.31***	-.04	.19***	-.03	-.01	.00	.03	-.07*	.02
2. Media use: Face-to-face	-	.29***	.28***	.48***	.32***	.12***	.11***	.12***	.13***	.21***	.14***
3. Media use: Newspaper		-	.38***	.30***	.35***	.03	.01	.09**	.04	.02	.09**
4. Media use: Television			-	.22***	.32***	.01	.01	.09*	.09*	.04	.06
5. Media use: Internet				-	.40***	.15***	.12***	.02	.03	.11**	.07
6. Political Interest					-	.17***	.15***	-.02	-.11**	-.06	.02
7. Ideological Strength: Linear						-	.93***	-.18***	-.11**	-.06	.04
8. Ideological Strength: Squared							-	-.16***	-.05	-.04	.08*
9. Ideological Self-Placement								-	.42***	.28***	.16***
10. Attitude toward asylum									-	.69***	.44***
11. Perceived Threat										-	.29***
12. Truly False Consensus											-

*Note*. Bivariate correlations for main variables.

* p < .05.

** p < .01.

*** p < .001.

**Table 3:** Distribution of mean scores across categories of information environments.

	Mixed	Neutral	Confirmative	Disconfirmative
Age	50.48 (16.68)	48.51 (17.24)	49.68 (15.03)	49.36 (16.11)
Media use				
Face-to-face	4.20 (1.16)	3.09 (1.29)	4.24 (1.17)	3.95 (1.12)
Newspaper	4.38 (1.61)	3.28 (1.81)	4.22 (1.76)	3.95 (1.73)
Television	5.14 (1.14)	4.34 (1.70)	5.06 (1.30)	4.86 (1.37)
Internet	4.37 (1.55)	3.22 (1.72)	4.26 (1.65)	4.23 (1.49)
Political interest	4.26 (1.27)	3.15 (1.44)	4.12 (1.39)	4.19 (1.36)
Ideological strength				
Linear	1.24 (1.30)	0.95 (1.15)	1.39 (1.49)	1.33 (1.23)
Squared	3.24 (4.95)	2.22 (3.91)	4.16 (6.57)	3.25 (4.93)
Ideological self-placement	5.64 (1.76)	5.59 (1.44)	6.24 (2.03)	5.27 (1.66)
Attitude towards asylum	3.92 (0.96)	3.90 (0.84)	4.66 (0.81)	3.52 (0.77)
Perceived threat	4.31 (1.23)	4.15 (1.23)	5.05 (1.10)	4.05 (1.19)
Truly False consensus	22.70 (22.03)	21.64 (20.87)	39.88 (25.28)	12.68 (17.42)

Note. Mean scores for main variables by information environment with standard deviation in parentheses.

### Antecedents of exposure to a confirmative information environment

To test our hypotheses H1, H2 and H3, we conducted multinomial logistic regression analyses with confirmative information environments as baseline category. All models included age, gender, work, formal education and political interest as control variables. Out of the latter, only political interest reached significance (see Table 4). It was a positive predictor of confirmative information environments, if compared to neutral information environments, in models 2a (OR = 0.63, p < .001), 2b (OR = 0.64, p < .001), and 3 (OR = 0.62, p < .001).

**Table 4:** Multinomial logistic regression analyses predicting information environments.

	Model 1			Model 2a			Model 2b			Model 3		
	Neutral	Mixed	Disconf.	Neutral	Mixed	Disconf.	Neutral	Mixed	Disconf.	Neutral	Mixed	Disconf.
Constant	9.55 (1.43)	0.33 (1.12)	0.72 (1.43)	5.85 (1.13)	0.43 (1.05)	0.42 (1.34)	7.17 (1.15)	0.48 (1.05)	0.49 (1.35)	31.97* (1.30)	2.06 (1.14)	3.27 (1.47)
Political interest	0.79 (0.11)	1.10 (0.09)	1.16 (0.12)	0.63** (0.10)	1.11 (0.08)	1.08 (0.11)	0.64** (0.10)	1.12 (0.08)	1.09 (0.11)	0.62** (0.10)	1.09 (0.08)	1.06 (0.11)
Face-to-face	0.63** (0.12)	0.94 (0.10)	0.80 (0.13)									
Newspaper	0.88 (0.09)	1.04 (0.07)	0.95 (0.09)									
Television	0.92 (0.10)	1.10 (0.09)	1.03 (0.11)									
Internet	1.00 (0.10)	1.00 (0.07)	1.04 (0.10)									
Extremism				0.91 (0.10)	0.97 (0.07)	1.01 (0.10)						
Ext. squared							0.96 (0.03)	0.98 (0.02)	0.98 (0.03)			
Ideological self-placem.										0.75** (0.08)	0.80** (0.06)	0.75** (0.08)
N	614			636			636			636		
McFadden R^2^	.33			.29			.29			.30		

Note. Results of multinomial logistic regressions for H1, H2 and H3 using confirmative information environments as baseline category. Cell entries show estimated odds ratios with standard errors in parentheses. Control variables were part of the analyses, but were excluded from presentation if they didn’t reach significance in any of the analyses.

* p < .01.

** p < .001.

In a first model, we tested how the use of different sources of information about the migration crisis predicted selective political exposure by adding face-to-face communication, Television, Newspaper and Internet use as independent predictors (see Table 4, Model 1). The technological hypothesis was not supported by our analysis. The frequency of using the Internet as source of information did not predict exposure to a confirmative information environment compared to the other information environments. Instead, face-to-face communication was the only significant predictor. The more participants talked about migration politics with friends and family, the higher the likelihood that they reported exposure to a confirmative information environment compared to a neutral information environment (OR = 0.63, p < .001). In other words, participants reporting exposure to a confirmative information environment were more likely to communicate face-to-face about the migration crisis compared to participants in a neutral environment (t = 8.64, p < .001, d = 0.94), but not compared to participants in a mixed (t = 0.49, p = .62, d = 0.04) and a disconfirmative information environment (t = 2.21, p = .03, d = 0.25), respectively.

Next, we tested the ideological strength hypothesis by using two different indicators for strength of ideological belief. A first estimator was calculated by centering the ideological self-placement item. Absolute values were used to approximate the strength of ideological belief, with the scale ranging from 0 (moderate) to 5 (extreme, see Table 4, Model 2a). We calculated a second estimator by using the square of the first estimator (see Table 4, Model 2b). Neither of the two measures reached significance in predicting selective political exposure. In other words, we found no empirical evidence for H2.

**Fig 2:**
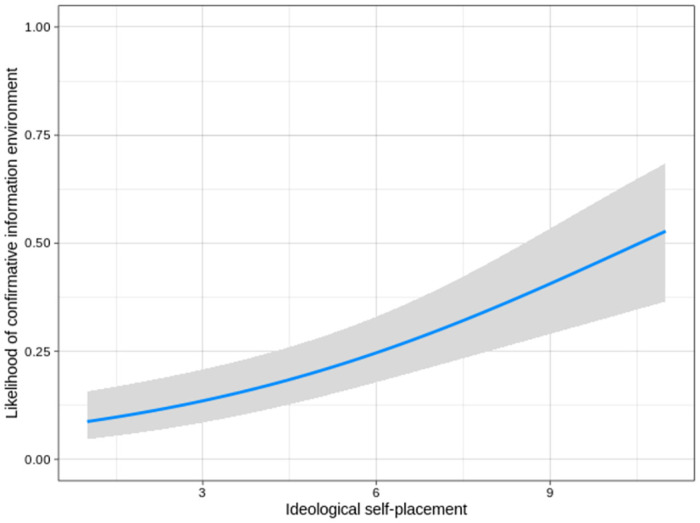
Likelihood of selective exposure by political ideology.

A linear effect of political conservatism on exposure to a confirmative information environment about migration politics was proposed by H3 (see Table 4, Model 3). Analyses provide support for this assumption. Political conservatism was a highly significant predictor of confirmative information environments compared to neutral (OR = 0.75, p < .001), mixed (OR = 0.80, p < .001) and disconfirmative information environments (OR = 0.75, p < .001). In other words, the more conservative a participant was in our sample, the higher the likelihood of him or her to be exposed to a confirmative information environment. This finding holds when we calculate pairwise comparisons with the other information environments. Participants reporting exposure to a confirmative information environment were more conservative compared to participants in a disconfirmative information environment (t = 4.70, p < .001, d = 0.52), participants in a neutral information environment (t = 3.62, p < .001, d = 0.37) and participants in a mixed information environment (t = 3.78, p < .001, d = 0.32). Fig 2 conveys an impression of the overall effect of conservatism on confirmative information environments if the latter are compared to the other information environments combined. While the likelihood of exposure to confirmative information environments is around 10% on the left side of the political ideology scale, it rises to more than 50% on its right.

Predicted likelihoods of confirmative information environments on the y-axis by ideological self-placement on the x-axis. The grey area represents a 95% confidence interval. The blue regression line is the result of a binary logistic regression that compared confirmative to other information environments via a dummy variable, controlling for age, gender, work and educational status as well as political interest.

The motivated social cognition framework used in H3 posits differences in the need for uncertainty avoidance and threat management at the core of partisan asymmetries. Consequently, we tested whether the relation between conservatism and selective political exposure was mediated by individual’s perception of threat. First, for each comparison, we tested the paths suggested by Baron and Kenny [55] for mediation analysis. The models for the direct effect were binary logistic regressions where confirmative were contrasted with the other information environments. In addition to conservatism and perceptions of threat, all control variables were included. Since only political interest had a significant effect in any of the paths considered, we excluded the remaining control variables from further analysis. In a next step, we used the *mediation* R package [56] to test for significance and size of the mediation. Bootstrapping procedures were used in 10,000 simulations of the respective models. In line with the theoretical reasoning, a significant proportion of the main direct effects of confirmative compared to neutral (b = .32, 95% CI [.14; .80], p = .003), mixed (b = .42, 95% CI [.22; .86], p < .001) and disconfirmative information environments (b = .29, 95% CI [.13; .58], p = .001) was mediated by perceptions of threat.

### Truly false consensus effect

We expected people with exposure to a confirmative information environment to report a stronger truly false consensus effect compared to people who are exposed to other information environments (H4). Our data supports this hypothesis (see Table 3 for Descriptives). Truly false consensus of participants communicating in a confirmative information environment was higher compared to participants in a mixed information environment (t = 8.53, p < .001, d = 0.73), a neutral information environment (t = 7.43, p < .001, d = 0.79) or a disconfirmative information environment (t = 11.66, p < .001, d = 1.25). Moreover, the truly false consensus effect in participants communicating in a disconfirmative information environment was lower compared to participants in a mixed information environment (t = -4.96, p < .001, d = -0.51) or a neutral information environment (t = -3.64, p < .001, d = -0.47). There was no difference in the truly false consensus effect between participants communicating in a mixed and a neutral information environment (t = 0.49, p = .62). Since our use of percentiles lead to a systematic variation of possible truly false consensus scores dependent on the relative position of an individual in the distribution of attitudes towards political asylum (see Discussion for details), we added a test to control for the influence of the relative distance of attitudes to the center of the distribution. We first conducted a linear regression to test if information environments were significant predictors of truly false consensus scores. This was the case, confirming our prior results. Disconfirmative (t = -3.11, p = .002, d = -0.22) as well as confirmative information environments (t = 7.51, p < .001, d = 0.52) were significant predictors of truly false consensus scores, while mixed and neutral information envirnoments were not. In a second analysis, we added a control variable indicating the relative distance of an individual’s attitude score to the center of the distribution. The control variable was calculated by subtracting the percentile indicating an individual’s position in the distribution of attitude scores from 50 and taking the absolute value. Adding this variable to the linear regression did not alter the pattern of results. While the control variable had a significant influence on false consensus scores (b = 0.90, p < .001, r = 0.55), its inclusion also heightened effect size and significance of disconfirmative information environments (t = -5.07, p < .001, d = -0.35). The influence of confirmative information environment (t = 7.36, p < .001, d = 0.51) more or less remained the same.

## Discussion

In the present study, we aimed to investigate potential antecedents and consequences of selective political exposure. By doing so, we tested predictions of prominent theories and assumptions about boundary conditions and motivational underpinnings of the phenomenon.

We found no empirical evidence that Internet use by itself fosters one-sided media diets. This finding is in conflict with the often articulated assumption that high-choice media environments generally lead to an increase in selective political exposure. Instead, our analyses support more modest assessments, arguing that “the relationship between selective exposure and the contemporary information environment is more complex” [57]. In addition to allowing for selective political exposure, Internet use may also foster accidental exposure to cross-cutting perspectives [3], [58]. For example, this can happen in social media or digital arenas, where politics is not frequently discussed [59]. There is also reason to believe that high-choice environments allow for the broadening of political perspectives. In other words, the Internet empowers individuals to approach and consume cross-cutting political information that might not be available in their personal communicative environment. In regard to the use of information channels, the only significant predictor of selective exposure in our data was face-to-face communication. This finding, which is in line with prior research [60, 61], not only contradicts the view that high-choice media environments are essentially responsible for one-sided media consumption, but strengthens the assumption that “individual choices” [6] rather than technological affordances are the most important predictors.

Focusing on these psychological predictors, our analyses support the assumption that conservatives are more inclined to selective political exposure compared to liberals. Since asymmetries between liberals and conservatives typically appear less pronounced if behavioral measures are used, as opposed to, for instance, self-report measures of cognitive constructs [62], our finding is significant. Obtained on the basis of individuals’, albeit self-reported, media use, this finding can be interpreted in line with the motivated cognition framework [16]. Based on this account, conservatives are generally more susceptible to threat and uncertainty. Additional support for this assumption comes from our analyses indicating that the relation between conservatism and selective political exposure was partially mediated by individuals’ perceptions of threat. Moreover, according to the motivated cognition framework [16], the experience of threat and uncertainty should promote closed-mindedness and a need for cognitive closure and, thus, make conservatives more susceptible to selective political exposure. Interpreted this way, our finding can point to a dispositional proclivity of conservatives to select confirmative information environments in order to reduce uncertainty. However, a more cautious interpretation should take into consideration that we focused on a specific political topic, namely political asylum and migration. Instead of drawing inferences about general preferences for selective political exposure, our findings might indicate that immigration is a highly threatening political topic for conservatives [38]. Based on this line of thinking, conservatives are especially likely to choose confirmative information environments when the immigration issue is at stake. In other words, there is reason to assume that conservatives tend to exchange or even form their political opinions about migration in politically homogenous information environments. This poses a challenge to the opposition of political polarization with regard to this highly controversial political topic. Given, as our analyses suggest, that perceptions of threat are key to this process, the growing number of refugees and migrants worldwide [63] might further amplify this tendency, since “[o]ne of the most consistent and enduring targets of right-wing criticism has been immigration, which is often experienced as frightening, confusing, and potentially threatening to the status quo” [16]. Knowing that perceived threat is linked to selective political exposure, it seems important to investigate in more detail how some political parties and agents use threatening communication in a strategic manner. For example, there is evidence that the communication of populists parties tends to be characterized by blaming others as the source of “crisis, breakdown, threat” [64]. In addition, certain media “intentionally or not […] play a particular role in spreading fear and cultivating perceptions of threat” [65].

We found no support for the assumption that selective exposure is generally fostered by the ideological strength of individuals’ belief systems. This finding challenges the assumption that avoidance of cognitive dissonance is the main psychological driver of selective political exposure. At present, cognitive dissonance theory [66] is still the “most commonly cited explanation” [12] for selective exposure—even though “the use of dissonance theory to specify particular circumstances under which selectivity would occur has not been a great success” [67]. In our case, the overly general motive of dissonance reduction seems badly qualified to explain partisan asymmetries. Such partisan asymmetries, however, are identified in a growing number of studies, including our own [7, 8, 34, 68]. Lately, the idea of a desire to share reality was introduced to explain partisan asymmetries in selective exposure. Motivated by the “pursuit of social verification and shared reality”, individuals are”moving to communities that reflect and reinforce their political values” [69]. Stronger for conservatives than for liberals [36, 70] and underpinning an individual’s “sense of certainty” [69], the desire to share reality is well suited to address the problem of partisan asymmetries from within the framework of motivated social cognition [16]. In addition to this relational motivation, the framework highlights partisan asymmetries in epistemic motivations like dogmatism, cognitive and perceptual rigidity, needs for order and structure, need for cognitive closure, intolerance of ambiguity and others [38]. Together, this set of motivations may provide a more suitable basis to explain partisan asymmetries in selective exposure than the need for dissonance avoidance.

In terms of the psychological consequences of selective exposure, we found empirical evidence for the relation with cognitive biases, that is, the truly false consensus effect. Individuals reporting selective political exposure inaccurately overestimated false consensus to a higher degree compared to individuals reporting exposure to other more diverse information environments. In other words, selective exposure was systematically linked to the tendency of individuals to inaccurately overestimate the amount of people with political attitudes similar to their own. To assess the truly false consensus effect, we devised a measure that differed in one main respect from measures typically used in such studies. This was necessary because we wanted to explore consensus on a continuous, as opposed to binary, variable. Typically, when pursuing the latter approach, consensus is operationalized as the amount of individuals endorsing an item relative to the total number of participants [17]. In practice, it means that participants either endorse an item, or they don’t, and then go on to estimate the share of others who did or did not endorse it. In contrast, we wanted to assess consensus with regard to attitudes towards political asylum, a continuous measure of sixteen items. On the basis of a continuous measure, however, the procedure to calculate consensus is no longer applicable. Instead of two antagonistic positions, we have attitudes scattered across the available spectrum. Participants therefore were not asked to estimate consensus itself, but their own position within this spectrum of really existing attitudes on political asylum. The estimation was then compared to the actual position in the sample and a truly false consensus effect thus assessed (see above). This revised procedure involved the use of percentiles to indicate the actual position of an individual which, in turn, led to systematically varied possible outcomes of the measure whereby those individuals at the opposite ends of the spectrum had possible values ranging from 0 to +/- 100 while the individual at the center of the distribution had possible values ranging from -50 to +50. We thank Russ Clay for this insight. The construction of our truly false consensus measure thus determined, to a certain extent, a V-shaped distribution of false consensus scores. Unfortunately, it is hard to say to *what* extent, especially since prior studies’ findings consistently support the expectation of such a distribution. As Mullen and Hu summarize their meta-analysis of studies on the false consensus effect, “The majority does significantly underestimate its consensus, the minority does significantly overestimate its consensus, and the minority’s overestimation is substantially stronger than the majority’s underestimation, giving rise to a significant false consensus effect” [71]. At any rate, the strongly asymmetrical distribution of truly false consensus scores (Fig 1) lead us to assume that, while the construction of our measure may have escalated effect sizes, it almost certainly did not bring them into being. In line with this, a post-hoc analysis revealed that the construction of the measure did not compromise our conclusions with regard to H4. The question remains, however, what kind of measure to use in future studies. We tried to devise a method for assessing truly false consensus effects with regard to topics that are more nuanced than a binary choice. This method, while useful in our particular study, unfortunately entailed new problems. Yet, whatever its shortcomings, we think that it is necessary to follow its direction and identify ways to investigate consensus in the context of complex societal issues irreducible to yes or no.

Our results were consequential with regard to the origin of a truly false consensus effect. Selective exposure has always been considered a possible source of a false consensus effect [46]. However, in this formative work, selective exposure was conceived as “nonmotivational factor” [46] based on availability. It thus referred to what was elsewhere called “de facto selectivity” [67]. Our results, on the other hand, do not support such an interpretation. First, none of the media investigated were associated with confirmative information envirnoments and thus selective exposure. Second, the fact that political ideology and conservatism, respectively, predict selective exposure is very hard to reconcile with “de facto selectivity,” since it would imply that media spread extremely, indeed the most negative views on asylum. Instead, our data suggest that perceptions of threat could be a motivational force behind selective exposure [40] and, consequently, behind an ensuing truly false consensus effect. The latter will likely form the basis for further bias. In a high-choice media environment with an abundant information supply, for instance, individuals are forced to rely on cognitive heuristics to judge the credibility or trustworthiness of a certain (online) source. A false consensus effect in such a scenario will likely strengthen a self-confirming heuristic [72] which, in turn, could strengthen the tendency to select confirmative information environments, itself a factor that might reinforce a false consensus effect. Such a mechanism could be described as a reinforcing spiral [73] where exogenous media effects are displaced by endogenous ones [74]. However problematic one may find such behavior, as *Argumentative Theory* points out, we should not treat it merely as a “*flaw of reasoning*” [75]. On the contrary, in particular situations, to engage in selective exposure, confirmation bias etc. should be understood as completely reasonable. To appreciate this, however, we need to get rid of the idea that it is, or should be, an individual’s goal at all times to reach a measured, well-justified opinion. Instead, in our context, we should expect individuals motivated by their perceptions of threat, anticipating discussion with others, trying to convince their interlocutors of their own perspective, not least to do away with the threat [75]. Applying this point of view allows us to take a different look at the sources of such behavior. To reiterate a point made above, it seems to us that, rather than being the result of technological changes like the introduction of the Internet, selective exposure and false consensus beliefs are dependent on individual’s motives and choices. Likewise, deciding whether or not it is reasonable, from an individual’s point of view, to engage in this kind of behavior, e.g., in the context of perceived threat, hardly involves a particular means of communication. This has consequences for possible interventions. For example, if our goal was to attenuate effects of selective exposure and false consensus beliefs, respectively, from this perspective, we should first look at individuals’ needs and motives. Unfortunately, our data, cross-sectional in nature, do not warrant such causal conclusions. Contrary to the lineup presented above, for instance, selective exposure could be a consequence of a strong false consensus effect, and not vice versa. To give the assumptions further credibility, studies are needed that allow for causal inferences.

This leads to the limitations of our study. As noted, our results are correlational and thus allow no claims on causality. In other words, we cannot rule out that alternative causal effects lead to the correlations that we observed in our data. For example, it is plausible that false consensus as a cognitive bias and selective political exposure are both the result of a common cause such as closed-mindedness. In order to provide more empirical support to the assumptions discussed in this paper, experimental studies are needed. Second, our investigation focused on communication about migration politics which cannot count as representative for political debate *per se*. As already mentioned, the topic of migration politics may be especially threatening to conservative individuals [16]. We should not generalize from our findings to other domains without testing this assumption empirically. Although there is some evidence for a general asymmetry in how liberals and conservatives engage in the processing of political information (for an overview see [38]), there is also research showing that these differences are highly dependent on political issues [76]. Finally, a more technical problem concerns the design of our response scales for items aiming at frequency. In the questions on information sources, for instance, we used ordinal, instead of interval-scaled, levels to assess people’s exposure to certain types of media. While we assumed that it would improve the reliability of such items if we used everyday differentiations (e.g., ‘every other week’, ‘weekly’, ‘a couple of times a week’ etc.), it certainly makes it more difficult to interpret odds ratios for each unit increase.

## Supporting information

S1 Text R script. Includes the data manipulations, analyses and plots used in the study.
(R) Click here for additional data file.

S2 Text Online questionnaire. The questionnaire includes the items we used as well as others not used in this particular study.
(PDF)

S1 Dataset Questionnaire data. The file includes respondents’ data in CSV format.
(CSV)
