# Supplementary material for: Conservative bias, selective political exposure and truly false consensus beliefs in political communication about the ‘refugee crisis’ in Germany
Source: PLoS One. 2021 Nov 4;16(11):e0259445. doi: 10.1371/journal.pone.0259445 (PMC8568288; doi:10.1371/journal.pone.0259445)
Supplement: S2 Text — The questionnaire includes the items we used as well as others not used in this particular study. (PDF) [file pone.0259445.s002.pdf]

UNIVERSITÄT MANNHEIM · UNIVERSITÄT KOBLENZ · LANDAU · TECHNISCHE UNIVERSITÄT DARMSTADT

Seite 01

Studie: Meinungen zur Flüchtlingskrise

Herzlich willkommen und vielen Dank für die Teilnahme an unserer Befragung.

In diesem Fragebogen interessieren wir uns dafür, aus welchen Gründen Menschen in der aktuellen Situation für bzw. gegen die Aufnahme von Flüchtlingen sind. Uns geht es **nicht** darum, persönliche Meinungen zu bewerten, sondern darum die Meinungen und Überzeugungen zur Flüchtlingskrise in der Bevölkerung realistisch abzubilden.

Die Bearbeitung des Fragebogens dauert 15 Minuten. Die Daten werden anonym erfasst und nur für wissenschaftliche Forschungszwecke ausgewertet.

Mit freundlichen Grüßen

Tobias Rothmund, Jun.-Prof. Dr.
Frank M. Schneider, Dr.
Diana Rieger, Dr.
Carsten Grimme, M.A.

PHP-Code

```
$id = readGET('i_survey');
replace('%panelID%', $id);

put('IV02_01', $id);
```

Seite 02

Sind Sie weiblich oder männlich?

- ○ weiblich
- ○ männlich

SD01 Geschlecht
1 = weiblich
2 = männlich
-9 = nicht beantwortet

Wie alt sind Sie? [ ]

SD02_01 Wie alt sind Sie?
Offene Eingabe (Ganze Zahl)

1. Was ist Ihr Schulabschluss?

- ○ kein Abschluss
- ○ noch in Ausbildung
- ○ Volks- oder Hauptschulabschluss
- ○ Real- oder Oberschulabschluss
- ○ Fachhochschule oder Hochschulreife

SD03 Schulabschluss
1 = kein Abschluss
2 = noch in Ausbildung
3 = Volks- oder Hauptschulabschluss
4 = Real- oder Oberschulabschluss
5 = Fachhochschule oder Hochschulreife
-9 = nicht beantwortet

Seite 03

PHP-Code

```
$age = value('SD02_01');
if ($age <= 24) {
  $agegroup = 1;
} elseif ($age <=34) {
  $agegroup = 2;
} elseif ($age <= 44) {
  $agegroup = 3;
} elseif ($age <=54) {
  $agegroup = 4;
} elseif ($age <=64) {
  $agegroup = 5;
} elseif ($age <=120) {
  $agegroup = 6;
}
put('IV01_01', $agegroup);


$gen = value('SD01');
$edu = value('SD03');
$n = statistic('crosscount', array('SD01','IV01_01','SD03'), array($gen, $agegroup, $edu));
debug($n);  // Nur aus Interesse

$groupID = 100 * $gen + 10 * $agegroup + $edu;
$limits = array(
111=> 2,
121=> 3,
131=>4,
141=>4,
151=>3,
161=>5,
112=>5,
122=>1,
113=>10,
123=>18,
133=>24,
143=>33,
153=>33,
163=>88,
114=>15,
124=>23,
134=>31,
144=>36,
154=>24,
164=>21,
115=>20,
125=>32,
135=>29,
145=>27,
155=>18,
165=>18,
211=>2,
221=>3,
231=>4,
241=>4,
251=>3,
261=>5,
212=>5,
222=>2,
213=>13,
223=>17,
233=>24,
243=>32,
253=>35,
263=>87,
214=>15,
224=>23,
234=>31,
244=>36,
254=>24,
264=>21,
215=>20,
225=>32,
235=>29,
245=>27,
255=>18,
265=>18
);
debug($limits[$groupID]);
if ($n >= $limits[$groupID]) {
  redirect('http://befragungen.keypanel.de/?m=6006&return=quotafull&securitykey=8TtpAFgfksEJexKMaxhZ&i_
}
```


Ihre Meinung zur Flüchtlingskrise

Auf den ersten Seiten geht es um Ihre persönliche Meinung zum Umgang mit der aktuellen Flüchtlingskrise.

2. Politikerinnen und Politiker fordern unterschiedliche Maßnahmen, um die aktuelle Flüchtlingskrise zu bewältigen. Wir haben hier einige zusammengetragen. Wie sehr stimmen Sie den einzelnen Forderungen zu?

Bitte geben Sie bei jeder Aussage an wie sehr Sie dieser persönlich zustimmen. Geben Sie Ihre Zustimmung an von *„stimme überhaupt nicht zu“* bis *„stimme voll und ganz zu“* ein.

	stimme überhaupt nicht zu					stimme voll und ganz zu
Kriminell gewordene Flüchtlinge sollten keine Chance auf Asyl haben und direkt abgeschoben werden.	○	○	○	○	○	○
Asylbewerber ohne einen Nachweis der Nationalität sollten in einem Schnellverfahren abgelehnt werden.	○	○	○	○	○	○
Die Zahl der Flüchtlinge, die in Deutschland aufgenommen werden, sollte begrenzt werden.	○	○	○	○	○	○
Flüchtlinge sollten uneingeschränkten Zugang zur Gesundheitsversorgung und den sozialen Sicherungssystemen erhalten.	○	○	○	○	○	○
Flüchtlinge sollten sofortigen Zugang zu Arbeit und Bildung erhalten.	○	○	○	○	○	○
Niemand darf in ein Land zurückgeschickt werden, in dem ihm Gefahr für Leib und Leben droht.	○	○	○	○	○	○
Asylbewerber mit geringer Bleibeperspektive sollten schneller in ihre Heimatländer zurückgeschickt werden können.	○	○	○	○	○	○
Es sollten „sichere“ Fluchtrouten für Flüchtlinge nach Europa eingerichtet werden.	○	○	○	○	○	○

GG02_01 Kriminell gewordene Flüchtlinge sollten keine Chance auf Asyl haben und direkt abgeschoben werden.
GG02_02 Asylbewerber ohne einen Nachweis der Nationalität sollten in einem Schnellverfahren abgelehnt werden.
GG02_03 Die Zahl der Flüchtlinge, die in Deutschland aufgenommen werden, sollte begrenzt werden.
GG02_04 Flüchtlinge sollten uneingeschränkten Zugang zur Gesundheitsversorgung und den sozialen Sicherungssystemen erhalten.
GG02_05 Flüchtlinge sollten sofortigen Zugang zu Arbeit und Bildung erhalten.
GG02_06 Niemand darf in ein Land zurückgeschickt werden, in dem ihm Gefahr für Leib und Leben droht.
GG02_07 Asylbewerber mit geringer Bleibeperspektive sollten schneller in ihre Heimatländer zurückgeschickt werden können.
GG02_08 Es sollten „sichere“ Fluchtrouten für Flüchtlinge nach Europa eingerichtet werden.

1 = stimme überhaupt nicht zu
6 = stimme voll und ganz zu
-9 = nicht beantwortet

3. Nun geht es um Fakten und Gerüchte im Zusammenhang mit der Flüchtlingskrise.

Im folgenden haben wir einige Aussagen und Behauptungen zusammengetragen. Inwiefern glauben Sie, dass die einzelnen Aussagen wahr sind?

Bitte geben Sie bei jeder Aussage an wie sehr Sie glauben, dass diese wahr ist. Schätzen Sie die Wahrheit von *„stimmt überhaupt nicht“* bis *„stimmt voll und ganz“* ein.

	stimmt überhaupt nicht					stimmt voll und ganz
An Flüchtlingsheime angrenzende Immobilien verlieren massiv an Wert.	○	○	○	○	○	○
Die Zahl der fremdenfeindlichen Übergriffe gegen Flüchtlinge ist im letzten Jahr stark gestiegen.	○	○	○	○	○	○
Flüchtlinge randalieren häufig und „vermüllen“ ihre Umgebung.	○	○	○	○	○	○
Die Flüchtlingsrouten werden von Terroristen zur illegalen Einreise nach Deutschland genutzt.	○	○	○	○	○	○

Flüchtlinge nehmen Einheimischen die Arbeitsplätze weg.	○	○	○	○	○	○
Straftaten von Flüchtlingen werden nicht verfolgt, die Polizei und der Staat schauen absichtlich weg.	○	○	○	○	○	○
Flüchtlinge bekommen mehr Geld als Hartz-IV-Empfänger.	○	○	○	○	○	○
Mindestens 30 Prozent der angeblichen Syrer, die als Flüchtlinge nach Deutschland kommen, sind gar keine.	○	○	○	○	○	○

GG01_01 An Flüchtlingsheime angrenzende Immobilien verlieren massiv an Wert.
GG01_02 Die Zahl der fremdenfeindlichen Übergriffe gegen Flüchtlinge ist im letzten Jahr stark gestiegen.
GG01_03 Flüchtlinge randalieren häufig und „vermüllen“ ihre Umgebung.
GG01_04 Die Flüchtlingsrouten werden von Terroristen zur illegalen Einreise nach Deutschland genutzt.
GG01_05 Flüchtlinge nehmen Einheimischen die Arbeitsplätze weg.
GG01_06 Straftaten von Flüchtlingen werden nicht verfolgt, die Polizei und der Staat schauen absichtlich weg.
GG01_07 Flüchtlinge bekommen mehr Geld als Hartz-IV-Empfänger.
GG01_08 Mindestens 30 Prozent der angeblichen Syrer, die als Flüchtlinge nach Deutschland kommen, sind gar keine.
1 = stimmt überhaupt nicht
6 = stimmt voll und ganz
-9 = nicht beantwortet

4. Wie hoch ist, nach Ihrer Einschätzung, der Anteil der Personen in der deutschen Gesellschaft, ...

... die **kritischer** gegenüber der Aufnahme von Flüchtlingen eingestellt sind als Sie?

Niemand / 0% Alle / 100%

... die **offener** gegenüber der Aufnahme von Flüchtlingen eingestellt sind als Sie?

Niemand / 0% Alle / 100%

AN01_01 ... die kritischer gegenüber der Aufnahme von Flüchtlingen eingestellt sind als Sie?
AN01_02 ... die offener gegenüber der Aufnahme von Flüchtlingen eingestellt sind als Sie?
1 = Niemand / 0%
101 = Alle / 100%
-9 = nicht beantwortet

5. Jeder macht sich andere Gedanken zur Flüchtlingskrise und deren Einfluss auf die Gesellschaft. Wie ist das bei Ihnen?

Bitte geben Sie bei jeder Aussage an wie sehr Sie zustimmen von *„stimme gar nicht zu“* bis *„stimme voll und ganz zu“* .

	stimme gar nicht zu					stimme voll und ganz zu
Ich mache mir Sorgen darüber wie unsere Gesellschaft mit der Flüchtlingskrise umgeht.	○	○	○	○	○	○
Ich befürchte negative Konsequenzen für unsere Gesellschaft als Folge der Flüchtlingskrise.	○	○	○	○	○	○
Je mehr ich über die Flüchtlingskrise nachdenke, desto besorgter bin ich um die Zukunft unserer Gesellschaft.	○	○	○	○	○	○

Ich finde, dass der Zusammenhalt unserer Gesellschaft durch die Flüchtlingskrise bedroht ist.	○	○	○	○	○	○
Eine „Willkommenskultur“ gegenüber Flüchtlingen löst bei mir Ärger aus.	○	○	○	○	○	○
Eine abweisende Haltung gegenüber Flüchtlingen löst bei mir Ärger aus.	○	○	○	○	○	○

PG02_01 Ich mache mir Sorgen darüber wie unsere Gesellschaft mit der Flüchtlingskrise umgeht.
PG02_02 Ich befürchte negative Konsequenzen für unsere Gesellschaft als Folge der Flüchtlingskrise.
PG02_04 Je mehr ich über die Flüchtlingskrise nachdenke, desto besorgter bin ich um die Zukunft unserer Gesellschaft.
PG02_05 Ich finde, dass der Zusammenhalt unserer Gesellschaft durch die Flüchtlingskrise bedroht ist.
PG02_06 Eine „Willkommenskultur“ gegenüber Flüchtlingen löst bei mir Ärger aus.
PG02_07 Eine abweisende Haltung gegenüber Flüchtlingen löst bei mir Ärger aus.

1 = stimme gar nicht zu
6 = stimme voll und ganz zu
-9 = nicht beantwortet

Seite 09

Wie und wo informieren Sie sich über die Flüchtlingskrise?

Nun geht es darum, wie und wo Sie sich über die Flüchtlingskrise informieren.

Seite 10

6. Wie häufig informieren Sie sich allgemein über politische Themen?

Bitte kreuzen Sie immer die Antwort an, die am ehesten auf Sie zutrifft.

Wie häufig haben Sie (sich) in den letzten 12 Monaten...

	nie	alle paar Monate	alle paar Wochen	wöchentl.	mehrmals in der Woche	täglich
... über das außenpolitische Geschehen in Europa bzw. der Welt informiert?	○	○	○	○	○	○
... über das innenpolitische Geschehen auf Bundes- bzw. Landesebene informiert?	○	○	○	○	○	○
... über das lokalpolitische Geschehen im Landkreis bzw. der Stadt oder der Gemeinde informiert?	○	○	○	○	○	○
... innerhalb des Familien- oder Bekanntenkreises Gespräche über politische Themen geführt?	○	○	○	○	○	○
... zu bestimmten politischen Themen gezielt nach Informationen gesucht (bspw. Im Internet, in Zeitungen oder Zeitschriften etc.)?	○	○	○	○	○	○

PE03_01 ... über das außenpolitische Geschehen in Europa bzw. der Welt informiert?
PE03_02 ... über das innenpolitische Geschehen auf Bundes- bzw. Landesebene informiert?
PE03_03 ... über das lokalpolitische Geschehen im Landkreis bzw. der Stadt oder der Gemeinde informiert?
PE03_04 ... innerhalb des Familien- oder Bekanntenkreises Gespräche über politische Themen geführt?
PE03_05 ... zu bestimmten politischen Themen gezielt nach Informationen gesucht (bspw. Im Internet, in Zeitungen oder Zeitschriften etc.)?

1 = nie
2 = alle paar Monate
3 = alle paar Wochen
4 = wöchentl.
5 = mehrmals in der Woche
6 = täglich
-9 = nicht beantwortet

7. Wie häufig informieren Sie sich über die Flüchtlingskrise...

	nie	alle paar Monate	alle paar Wochen	wöchentl.	mehrmals in der Woche	täglich
... im Fernsehen?	○	○	○	○	○	○
... in Zeitungen?	○	○	○	○	○	○
... im Internet?	○	○	○	○	○	○
... in Gesprächen mit Freunden und Bekannten?	○	○	○	○	○	○

NK01_01 ... im Fernsehen?
NK01_02 ... in Zeitungen?
NK01_03 ... im Internet?
NK01_04 ... in Gesprächen mit Freunden und Bekannten?
1 = nie
2 = alle paar Monate
3 = alle paar Wochen
4 = wöchentl.
5 = mehrmals in der Woche
6 = täglich
-9 = nicht beantwortet

8. Wie häufig hören/lesen Sie Aussagen von Menschen, die ...

	nie	alle paar Monate	alle paar Wochen	wöchentl.	mehrmals in der Woche	täglich
... sich stark gegen die Aufnahme von Flüchtlingen aussprechen?	○	○	○	○	○	○
... unentschieden sind, was die Aufnahme von Flüchtlingen angeht?	○	○	○	○	○	○
... sich stark für die Aufnahme von Flüchtlingen aussprechen?	○	○	○	○	○	○

PG01_01 ... sich stark gegen die Aufnahme von Flüchtlingen aussprechen?
PG01_02 ... unentschieden sind, was die Aufnahme von Flüchtlingen angeht?
PG01_03 ... sich stark für die Aufnahme von Flüchtlingen aussprechen?
1 = nie
2 = alle paar Monate
3 = alle paar Wochen
4 = wöchentl.
5 = mehrmals in der Woche
6 = täglich
-9 = nicht beantwortet

9. Haben Sie einen Facebook Account?

○ Ja
○ Nein

○ Ich bin nicht sicher

F001 Nutzen Sie Facebook?
1 = Ja
2 = Nein
-1 = Ich bin nicht sicher
-9 = nicht beantwortet

PHP-Code

```
if (value('F001') == 2) {
  goToPage('keinfb');
}
```

PHP-Code

```
if (value('F001') == -1) {
  goToPage('keinfb');
}
```

Seite 15

10. An einem durchschnittlichen Tag, wie viel Zeit verbringen Sie auf Facebook?

Gar keine Zeit				Ungefähr eine Stunde				Ungefähr zwei Stunden				Drei Stunden und mehr
○	○	○	○	○	○	○	○	○	○	○	○	○

FB01 Facebook Nutzungsdauer
1 = Gar keine Zeit [1]
2 = [2]
3 = [3]
4 = [4]
5 = Ungefähr eine Stunde [5]
6 = [6]
7 = [7]
8 = [8]
9 = Ungefähr zwei Stunden [9]
10 = [10]
11 = [11]
12 = [12]
13 = Drei Stunden und mehr [13]
-9 = nicht beantwortet

Seite 16

11. Wie oft lesen Sie auf Facebook ...

	nie	alle paar Monate	alle paar Wochen	wöchentl.	mehrmals in der Woche	täglich
... Nachrichten über die Flüchtlingskrise?	○	○	○	○	○	○
... persönliche Kommentare über die Flüchtlingskrise?	○	○	○	○	○	○
... persönliche Kommentare von Menschen, die sich **für** die Aufnahme von Flüchtlingen aussprechen?	○	○	○	○	○	○
... persönliche Kommentare von Menschen, die sich **gegen** die Aufnahme von Flüchtlingen aussprechen?	○	○	○	○	○	○
... persönliche Kommentare von Menschen, in denen Andere beleidigt werden?	○	○	○	○	○	○

NK02_01 ... Nachrichten über die Flüchtlingskrise?
NK02_02 ... persönliche Kommentare über die Flüchtlingskrise?
NK02_03 ... persönliche Kommentare von Menschen, die sich für die Aufnahme von Flüchtlingen aussprechen?
NK02_04 ... persönliche Kommentare von Menschen, die sich gegen die Aufnahme von Flüchtlingen aussprechen?
NK02_05 ... persönliche Kommentare von Menschen, in denen Andere beleidigt werden?
1 = nie
2 = alle paar Monate
3 = alle paar Wochen
4 = wöchentl.
5 = mehrmals in der Woche
6 = täglich
-9 = nicht beantwortet

Politisches Engagement

Jetzt geht es darum, wie sehr Sie sich selbst politisch engagieren.

12. Menschen unterscheiden sich darin, wie häufig und in welcher Form Sie am politischen Leben teilnehmen. Bei den folgenden Fragen geht es darum, in welcher Art und Weise Sie politisch aktiv sind.

Bitte kreuzen Sie immer die Antwort an, die am ehesten auf Sie zutrifft.

Wie häufig haben Sie (sich) in den letzten 12 Monaten...

	nie	selten	manchmal	oft	sehr oft
... aus sozialen, politischen, oder ethischen Gründen ein Produkt gezielt gekauft bzw. gezielt nicht gekauft? (bspw. fair gehandelte Waren etc.)	○	○	○	○	○
... eine Unterschriftenliste zu einem politischen Thema unterschrieben?	○	○	○	○	○
... an einer genehmigten politischen Veranstaltung teilgenommen (bspw. Kundgebung, Demonstration etc.)?	○	○	○	○	○
... an der Verbreitung politischer Informationen beteiligt (bspw. Flyer, Plakate, Dokumente, Dateien, Internetlinks etc.)	○	○	○	○	○
... aktiv in einer sozialen oder politischen Organisation oder Bewegung mitgearbeitet (bspw. Partei, Bürgerinitiative, Stiftung, Gewerkschaft, Kirche, Nichtregierungsorganisation, Verein etc.)?	○	○	○	○	○

PE04_01 ... aus sozialen, politischen, oder ethischen Gründen ein Produkt gezielt gekauft bzw. gezielt nicht gekauft? (bspw. fair gehandelte Waren etc.)
PE04_02 ... eine Unterschriftenliste zu einem politischen Thema unterschrieben?
PE04_03 ... an einer genehmigten politischen Veranstaltung teilgenommen (bspw. Kundgebung, Demonstration etc.)?
PE04_04 ... an der Verbreitung politischer Informationen beteiligt (bspw. Flyer, Plakate, Dokumente, Dateien, Internetlinks etc.)
PE04_05 ... aktiv in einer sozialen oder politischen Organisation oder Bewegung mitgearbeitet (bspw. Partei, Bürgerinitiative, Stiftung, Gewerkschaft, Kirche, Nichtregierungsorganisation, Verein etc.)?

1 = nie
2 = selten
3 = manchmal
4 = oft
5 = sehr oft
-9 = nicht beantwortet

PHP-Code

```
if (value('F001') == 2) {
  goToPage('keinfb2');
}
```

PHP-Code

```
if (value('F001') == -1) {
  goToPage('keinfb2');
}
```


13. Bei den folgenden Fragen geht es darum, in welcher Art und Weise Sie in Bezug auf die Flüchtlingskrise aktiv auf Facebook waren.

Bitte kreuzen Sie immer die Antwort an, die am ehesten auf Sie zutrifft.

Wie häufig haben Sie in den letzten 12 Monaten...

	nie	alle paar Monate	alle paar Wochen	wöchentl.	mehrmals in der Woche	täglich
... Informationen zur Flüchtlingskrise auf Facebook geteilt?	○	○	○	○	○	○
... die eigene Meinung zur Flüchtlingskrise auf Facebook geäußert?	○	○	○	○	○	○
... andere Beitrage zur Flüchtlingskrise auf Facebook kommentiert?	○	○	○	○	○	○
... Beiträge zur Flüchtlingskrise geliked?	○	○	○	○	○	○

PE05_01 ... Informationen zur Flüchtlingskrise auf Facebook geteilt?
PE05_02 ... die eigene Meinung zur Flüchtlingskrise auf Facebook geäußert?
PE05_03 ... andere Beitrage zur Flüchtlingskrise auf Facebook kommentiert?
PE05_04 ... Beiträge zur Flüchtlingskrise geliked?

1 = nie
2 = alle paar Monate
3 = alle paar Wochen
4 = wöchentl.
5 = mehrmals in der Woche
6 = täglich
-9 = nicht beantwortet

14. Sind Sie in einer Facebookgruppe die sich <u>für</u> die Aufnahme von Flüchtlingen einsetzt? Wenn ja, ungefähr seit wie vielen Wochen/Monaten?

[Bitte auswählen]

FG01 Facebook Gruppe PRO
20 = Nein, ich bin in keiner solchen Gruppe
1 = 1 Woche
2 = 2 Wochen
3 = 3 Wochen
4 = 4 Wochen
5 = 5 Wochen
6 = 6 Wochen
7 = 7 Wochen
8 = 8 Wochen
9 = über 2 Monate
10 = über 3 Monate
11 = über 4 Monate
12 = über 5 Monate
13 = über 6 Monate
14 = über 7 Monate
15 = über 8 Monate
16 = über 9 Monate
17 = über 10 Monate
18 = über 11 Monate
19 = länger als 1 Jahr
-9 = nicht beantwortet

15. Sind Sie in einer Facebookgruppe die sich <u>gegen</u> die Aufnahme von Flüchtlingen einsetzt? Wenn ja, ungefähr seit wie vielen Wochen?

[Bitte auswählen]

FG02 Facebook Gruppe CONTRA
9 = Nein, ich bin in keiner solchen Gruppe
1 = 1 Woche
2 = 2 Wochen
3 = 3 Wochen
4 = 4 Wochen
5 = 5 Wochen
6 = 6 Wochen
7 = 7 Wochen
8 = 8 Wochen
10 = über 2 Monate
11 = über 3 Monate
12 = über 4 Monate
13 = über 5 Monate
14 = über 6 Monate
15 = über 7 Monate
16 = über 8 Monate
17 = über 9 Monate
18 = über 10 Monate
19 = über 11 Monate
20 = länger als 1 Jahr
-1 = nein
-9 = nicht beantwortet

keinfb2

16. Haben Sie sich in den letzten 12 Monaten außerhalb des Internets für oder gegen Flüchtlinge engagiert?

○ Ich habe mich für die Aufnahme von Flüchtlingen engagiert (bspw. Geld gespendet, Deutsch unterrichtet).

○ Ich habe mich gegen die Aufnahme von Flüchtlingen engagiert (bspw. demonstriert, Informationsmaterial verteilt).

○ Ich habe nichts dergleichen getan.

FI01 Filter Pro/Kontra Flüchtlinge Engagement
1 = Ich habe mich für die Aufnahme von Flüchtlingen engagiert (bspw. Geld gespendet, Deutsch unterrichtet).
2 = Ich habe mich gegen die Aufnahme von Flüchtlingen engagiert (bspw. demonstriert, Informationsmaterial verteilt).
-1 = Ich habe nichts dergleichen getan.
-9 = nicht beantwortet

PHP-Code

```
if (value('FI01') == 2) {
  goToPage('contra');
}
```

PHP-Code

```
if (value('FI01') == -1) {
  goToPage('weiter');
}
```


pro

PHP-Code

```
prepare_input('ZE01_01');
```

17. Bei den folgenden Fragen geht es darum, in welcher Art und Weise Sie sich für Flüchtlinge eingesetzt haben.

Bitte kreuzen Sie immer die Antwort an, die am ehesten auf Sie zutrifft.

Wie häufig haben Sie in den letzten 12 Monaten...

	nie	alle paar Monate	alle paar Wochen	wöchentl.	mehrmals in der Woche	täglich
... Geld gespendet?	○	○	○	○	○	○
... Deutsch unterrichtet?	○	○	○	○	○	○
... Begleitung zu Ämtern und Hilfe bei Anträgen geleistet?	○	○	○	○	○	○
... Wohnraum zur Verfügung gestellt?	○	○	○	○	○	○
... Freizeitbeschäftigungen für Flüchtlinge angeboten?	○	○	○	○	○	○
... Kleider- und Sachspenden gegeben?	○	○	○	○	○	○
Sonstiges: ⇉ ZE01_01 ⇇	○	○	○	○	○	○

PE06_01 ... Geld gespendet?
PE06_02 ... Deutsch unterrichtet?
PE06_03 ... Begleitung zu Ämtern und Hilfe bei Anträgen geleistet?
PE06_04 ... Wohnraum zur Verfügung gestellt?
PE06_05 ... Freizeitbeschäftigungen für Flüchtlinge angeboten?
PE06_06 ... Kleider- und Sachspenden gegeben?
PE06_07 Sonstiges: %ZE01_01%

1 = nie
2 = alle paar Monate
3 = alle paar Wochen
4 = wöchentl.
5 = mehrmals in der Woche
6 = täglich
-9 = nicht beantwortet

Seite 25

PHP-Code

```
if (value('FI01') == 1) {
  goToPage('weiter');
}
```

Seite 26

contra

PHP-Code

```
prepare_input('ZE01_02');
```

18. Bei den folgenden Fragen geht es darum, in welcher Art und Weise Sie sich gegen Flüchtlinge eingesetzt haben.

Bitte kreuzen Sie immer die Antwort an, die am ehesten auf Sie zutrifft.

Wie häufig haben Sie in den letzten 12 Monaten...

	nie	alle paar Monate	alle paar Wochen	wöchentl.	mehrmals in der Woche	täglich
... Geld für Organisationen gespendet, die sich gegen die Aufnahme von Flüchtlingen einsetzen?	○	○	○	○	○	○
... an Demonstrationen gegen Flüchtlinge teilgenommen?	○	○	○	○	○	○
... Informationsmaterialien (Flyer, Broschüren, etc.) gegen Flüchtlinge verteilt?	○	○	○	○	○	○
... andere von ihrer Meinung gegen Flüchtlinge zu überzeugen versucht?	○	○	○	○	○	○
... eine Bürgerinitiative gegen die Errichtung eines Flüchtlingsheims unterstützt?	○	○	○	○	○	○
... Informationsveranstaltungen gegen Flüchtlinge besucht?	○	○	○	○	○	○
Sonstiges: ⇉ ZE01_02 ⇇	○	○	○	○	○	○

PE07_01 ... Geld für Organisationen gespendet, die sich gegen die Aufnahme von Flüchtlingen einsetzen?
PE07_02 ... an Demonstrationen gegen Flüchtlinge teilgenommen?
PE07_03 ... Informationsmaterialien (Flyer, Broschüren, etc.) gegen Flüchtlinge verteilt?
PE07_04 ... andere von ihrer Meinung gegen Flüchtlinge zu überzeugen versucht?
PE07_05 ... eine Bürgerinitiative gegen die Errichtung eines Flüchtlingsheims unterstützt?
PE07_06 ... Informationsveranstaltungen gegen Flüchtlinge besucht?
PE07_07 Sonstiges: %ZE01_02%

1 = nie
2 = alle paar Monate
3 = alle paar Wochen
4 = wöchentl.
5 = mehrmals in der Woche
6 = täglich
-9 = nicht beantwortet

Seite 27

weiter

Persönlichkeit und politische Einstellungen

Abschließend geht es um Ihre Person und Ihre allgemeinen politischen Einstellungen.

Seite 28

19. Menschen reagieren in unfairen Situationen sehr unterschiedlich. Wie ist es bei Ihnen? Zunächst geht es um Situationen, die zum Vorteil anderer und zu Ihrem Nachteil ausgehen.

	trifft überhaupt nicht zu					trifft voll und ganz zu
Es ärgert mich, wenn es anderen unverdient besser geht als mir.	○	○	○	○	○	○
Es macht mir zu schaffen, wenn ich mich für Dinge abrackern muss, die anderen in den Schoß fallen.	○	○	○	○	○	○

UG01_01 Es ärgert mich, wenn es anderen unverdient besser geht als mir.
UG01_02 Es macht mir zu schaffen, wenn ich mich für Dinge abrackern muss, die anderen in den Schoß fallen.
1 = trifft überhaupt nicht zu
6 = trifft voll und ganz zu
-9 = nicht beantwortet

Seite 29

20. Nun geht es um Situationen, in denen Sie mitbekommen oder erfahren haben, dass jemand anderes unfair behandelt, benachteiligt oder ausgenutzt wird.

	trifft überhaupt nicht zu					trifft voll und ganz zu
Ich bin empört, wenn es jemandem unverdient schlechter geht als anderen.	○	○	○	○	○	○
Es macht mir zu schaffen, wenn sich jemand für Dinge abrackern muss, die anderen in den Schoß fallen.	○	○	○	○	○	○

UG02_01 Ich bin empört, wenn es jemandem unverdient schlechter geht als anderen.
UG02_02 Es macht mir zu schaffen, wenn sich jemand für Dinge abrackern muss, die anderen in den Schoß fallen.
1 = trifft überhaupt nicht zu
6 = trifft voll und ganz zu
-9 = nicht beantwortet

Seite 30

21. Nun geht es um Ihre allgemeine Lebenszufriedenheit. Wie zufrieden sind Sie gegenwärtig, alles in allem, mit Ihrem Leben?

○	○	○	○	○	○	○	○	○	○	○
überhaupt nicht zufrieden	1	2	3	4	5	6	7	8	9	völlig zufrieden

LB01 Lebenszufriedenheit
1 = überhaupt nicht zufrieden
2 = 1
3 = 2
4 = 3
5 = 4
6 = 5
7 = 6
8 = 7
9 = 8
10 = 9
11 = völlig zufrieden
-9 = nicht beantwortet

Seite 31

22. Wie beurteilen Sie die folgenden Aussagen?

	stimme ganz und gar nicht zu				stimme voll und ganz zu
Gegen Außenseiter und Nichtstuer sollte in der Gesellschaft mit aller Härte vorgegangen werden.	○	○	○	○	○
Unruhestifter sollten deutlich zu spüren bekommen, dass sie in der Gesellschaft unerwünscht sind.	○	○	○	○	○
Gesellschaftliche Regeln sollten ohne Mitleid durchgesetzt werden.	○	○	○	○	○
Wir brauchen starke Führungspersonen damit wir in der Gesellschaft sicher leben können.	○	○	○	○	○
Menschen sollten wichtige Entscheidungen in der Gesellschaft Führungspersonen überlassen.	○	○	○	○	○
Wir sollten dankbar sein für führende Köpfe, die uns genau sagen, was wir tun können.	○	○	○	○	○
Traditionen sollten unbedingt gepflegt und aufrechterhalten werden.	○	○	○	○	○
Bewährte Verhaltensweisen sollten nicht in Frage gestellt werden.	○	○	○	○	○
Es ist immer das Beste, Dinge in der üblichen Art und Weise zu machen.	○	○	○	○	○

SO01_01 Gegen Außenseiter und Nichtstuer sollte in der Gesellschaft mit aller Härte vorgegangen werden.
SO01_02 Unruhestifter sollten deutlich zu spüren bekommen, dass sie in der Gesellschaft unerwünscht sind.
SO01_03 Gesellschaftliche Regeln sollten ohne Mitleid durchgesetzt werden.
SO01_04 Wir brauchen starke Führungspersonen damit wir in der Gesellschaft sicher leben können.
SO01_05 Menschen sollten wichtige Entscheidungen in der Gesellschaft Führungspersonen überlassen.
SO01_06 Wir sollten dankbar sein für führende Köpfe, die uns genau sagen, was wir tun können.
SO01_07 Traditionen sollten unbedingt gepflegt und aufrechterhalten werden.
SO01_08 Bewährte Verhaltensweisen sollten nicht in Frage gestellt werden.
SO01_09 Es ist immer das Beste, Dinge in der üblichen Art und Weise zu machen.

1 = stimme ganz und gar nicht zu
5 = stimme voll und ganz zu
-9 = nicht beantwortet

---

23. Im Folgenden finden Sie eine Reihe von Aussagen darüber, in welcher Beziehung gesellschaftliche Gruppen zueinander stehen sollten. Gesellschaftliche Gruppen können dabei z.B. ethnische Gruppen, politische Gruppen, religiöse Gruppen, Berufsgruppen oder auch die beiden Geschlechter sein.

Bitte geben Sie an, wie stark Sie persönlich den Aussagen zustimmen.

	stimme überhaupt nicht zu			teils, teils			stimme voll und ganz zu
Um das zu bekommen, was man möchte, ist es manchmal notwendig, Härte gegenüber anderen Gruppen zu zeigen.	○	○	○	○	○	○	○
Um im Leben voranzukommen, ist es manchmal nötig, keine Rücksicht auf andere Gruppen zu nehmen.	○	○	○	○	○	○	○
Manche Gruppen haben mehr Chancen im Leben als andere, das ist völlig in Ordnung so.	○	○	○	○	○	○	○
Gruppengleichheit sollte unser Ideal sein.	○	○	○	○	○	○	○
Alle Gruppen sollten die gleichen Chancen im Leben haben.	○	○	○	○	○	○	○
Es wäre gut, wenn alle Gruppen gleichgestellt wären.	○	○	○	○	○	○	○

GP01_02 Um das zu bekommen, was man möchte, ist es manchmal

notwendig, Härte gegenüber anderen Gruppen zu zeigen.
GP01_05 Um im Leben voranzukommen, ist es manchmal nötig, keine Rücksicht auf andere Gruppen zu nehmen.
GP01_06 Manche Gruppen haben mehr Chancen im Leben als andere, das ist völlig in Ordnung so.
GP01_07 Gruppengleichheit sollte unser Ideal sein.
GP01_08 Alle Gruppen sollten die gleichen Chancen im Leben haben.
GP01_09 Es wäre gut, wenn alle Gruppen gleichgestellt wären.

1 = stimme überhaupt nicht zu
2 =
3 =
4 = teils, teils
5 =
6 =
7 = stimme voll und ganz zu
-9 = nicht beantwortet

Seite 33

Bitte geben Sie an, wie stark Sie persönlich den Aussagen zustimmen.

	stimme überhaupt nicht zu			teils, teils			stimme voll und ganz zu
Gruppengleichheit sollte unser Ideal sein.	○	○	○	○	○	○	○
Es ist wahrscheinlich ganz gut, dass bestimmte Gruppen in der Gesellschaft oben stehen und andere unten.	○	○	○	○	○	○	○
Alle Gruppen sollten die gleichen Chancen im Leben haben.	○	○	○	○	○	○	○
Soziale Gleichheit sollte zunehmen.	○	○	○	○	○	○	○
Unterlegene Gruppen sollten unter sich bleiben.	○	○	○	○	○	○	○
Es wäre gut, wenn alle Gruppen gleichgestellt wären.	○	○	○	○	○	○	○

GP02_01 Gruppengleichheit sollte unser Ideal sein.
GP02_02 Es ist wahrscheinlich ganz gut, dass bestimmte Gruppen in der Gesellschaft oben stehen und andere unten.
GP02_03 Alle Gruppen sollten die gleichen Chancen im Leben haben.
GP02_04 Soziale Gleichheit sollte zunehmen.
GP02_05 Unterlegene Gruppen sollten unter sich bleiben.
GP02_06 Es wäre gut, wenn alle Gruppen gleichgestellt wären.

1 = stimme überhaupt nicht zu
2 =
3 =
4 = teils, teils
5 =
6 =
7 = stimme voll und ganz zu
-9 = nicht beantwortet

Seite 34

PHP-Code

```
if (value('SD01') == 2) {
  goToPage('valmann');
}
```

Seite 35

valfrau

24. Im Folgenden beschreiben wir Ihnen einige Personen. Bitte lesen Sie jede Beschreibung und überlegen Sie, wie sehr Ihnen diese Person ähnelt oder nicht ähnelt.

Bitte kreuzen Sie an, wie ähnlich oder unähnlich Ihnen die jeweils beschriebene Person ist.

	ist mir überhaupt nicht ähnlich					ist mir sehr ähnlich
Es ist ihr wichtig, neue Ideen zu entwickeln und kreativ zu sein. Sie macht Sachen gerne auf ihre eigene originelle Art und Weise.	○	○	○	○	○	○
Es ist ihr wichtig, reich zu sein. Sie möchte viel Geld haben und teure Sachen besitzen.	○	○	○	○	○	○

Sie hält es für wichtig, dass alle Menschen auf der Welt gleich behandelt werden sollten. Sie glaubt, dass jeder Mensch im Leben gleiche Chancen haben sollte.	○	○	○	○	○	○
Es ist ihr wichtig, ihre Fähigkeiten zu zeigen. Sie möchte, dass die Leute bewundern, was sie tut.	○	○	○	○	○	○
Es ist ihr wichtig, in einem sicheren Umfeld zu leben. Sie vermeidet alles, was ihre Sicherheit gefährden könnte.	○	○	○	○	○	○
Sie mag Überraschungen und hält immer Ausschau nach neuen Aktivitäten. Sie denkt, dass im Leben Abwechslung wichtig ist.	○	○	○	○	○	○
Sie glaubt, dass die Menschen tun sollten, was man Ihnen sagt. Sie denkt, dass Menschen sich immer an Regeln halten sollten, selbst dann wenn es niemand sieht.	○	○	○	○	○	○
Es ist ihr wichtig, Menschen zuzuhören, die anders sind als sie. Auch wenn sie anderer Meinung ist als andere, will sie die anderen trotzdem verstehen.	○	○	○	○	○	○

PV01_01 Es ist ihr wichtig, neue Ideen zu entwickeln und kreativ zu sein. Sie macht Sachen gerne auf ihre eigene originelle Art und Weise.
PV01_02 Es ist ihr wichtig, reich zu sein. Sie möchte viel Geld haben und teure Sachen besitzen.
PV01_03 Sie hält es für wichtig, dass alle Menschen auf der Welt gleich behandelt werden sollten. Sie glaubt, dass jeder Mensch im Leben gleiche Chancen haben sollte.
PV01_04 Es ist ihr wichtig, ihre Fähigkeiten zu zeigen. Sie möchte, dass die Leute bewundern, was sie tut.
PV01_05 Es ist ihr wichtig, in einem sicheren Umfeld zu leben. Sie vermeidet alles, was ihre Sicherheit gefährden könnte.
PV01_06 Sie mag Überraschungen und hält immer Ausschau nach neuen Aktivitäten. Sie denkt, dass im Leben Abwechslung wichtig ist.
PV01_07 Sie glaubt, dass die Menschen tun sollten, was man Ihnen sagt. Sie denkt, dass Menschen sich immer an Regeln halten sollten, selbst dann wenn es niemand sieht.
PV01_08 Es ist ihr wichtig, Menschen zuzuhören, die anders sind als sie. Auch wenn sie anderer Meinung ist als andere, will sie die anderen trotzdem verstehen.

1 = ist mir überhaupt nicht ähnlich
6 = ist mir sehr ähnlich
-9 = nicht beantwortet

Seite 36

Bitte kreuzen Sie an, wie ähnlich oder unähnlich Ihnen die jeweils beschriebene Person ist.

	ist mir überhaupt nicht ähnlich					ist mir sehr ähnlich
Es ist ihr wichtig, zurückhaltend und bescheiden zu sein. Sie versucht, die Aufmerksamkeit nicht auf sich zu lenken.	○	○	○	○	○	○
Es ist ihr wichtig, Spaß zu haben. Sie gönnt sich selbst gerne etwas.	○	○	○	○	○	○
Es ist ihr wichtig, selbst zu entscheiden, was sie tut. Sie ist gerne frei und unabhängig von anderen.	○	○	○	○	○	○
Es ist ihr sehr wichtig, den Menschen um sie herum zu helfen. Sie will für deren Wohl sorgen.	○	○	○	○	○	○
Es ist ihr wichtig, sehr erfolgreich zu sein. Sie hofft, dass die Leute ihre Leistungen anerkennen.	○	○	○	○	○	○
Es ist ihr wichtig, dass der Staat ihre persönliche Sicherheit vor allen Bedrohungen gewährleistet. Sie will einen starken Staat, der seine Bürger verteidigt.	○	○	○	○	○	○
Sie sucht das Abenteuer und geht gerne Risiken ein. Sie will ein aufregendes Leben haben.	○	○	○	○	○	○
Es ist ihr wichtig, sich jederzeit korrekt zu verhalten. Sie vermeidet es, Dinge zu tun, die andere Leute für falsch halten könnten.	○	○	○	○	○	○

PV02_01 Es ist ihr wichtig, zurückhaltend und bescheiden zu sein. Sie versucht, die Aufmerksamkeit nicht auf sich zu lenken.
PV02_02 Es ist ihr wichtig, Spaß zu haben. Sie gönnt sich selbst gerne etwas.

PV02_03 Es ist ihr wichtig, selbst zu entscheiden, was sie tut. Sie ist gerne frei und unabhängig von anderen.
PV02_04 Es ist ihr sehr wichtig, den Menschen um sie herum zu helfen. Sie will für deren Wohl sorgen.
PV02_05 Es ist ihr wichtig, sehr erfolgreich zu sein. Sie hofft, dass die Leute ihre Leistungen anerkennen.
PV02_06 Es ist ihr wichtig, dass der Staat ihre persönliche Sicherheit vor allen Bedrohungen gewährleistet. Sie will einen starken Staat, der seine Bürger verteidigt.
PV02_07 Sie sucht das Abenteuer und geht gerne Risiken ein. Sie will ein aufregendes Leben haben.
PV02_08 Es ist ihr wichtig, sich jederzeit korrekt zu verhalten. Sie vermeidet es, Dinge zu tun, die andere Leute für falsch halten könnten.

1 = ist mir überhaupt nicht ähnlich
6 = ist mir sehr ähnlich
-9 = nicht beantwortet

Bitte kreuzen Sie an, wie ähnlich oder unähnlich Ihnen die jeweils beschriebene Person ist.

	ist mir überhaupt nicht ähnlich					ist mir sehr ähnlich
Es ist ihr wichtig, dass andere sie respektieren. Sie will, dass die Leute tun, was sie sagt.	○	○	○	○	○	○
Es ist ihr wichtig, ihren Freunden gegenüber loyal zu sein. Sie will sich für Menschen einsetzen, die ihr nahe stehen.	○	○	○	○	○	○
Sie ist fest davon überzeugt, dass die Menschen sich um die Natur kümmern sollten. Umweltschutz ist ihr wichtig.	○	○	○	○	○	○
Tradition ist ihr wichtig. Sie versucht, sich an die Sitten und Gebräuche zu halten, die ihr von ihrer Religion oder ihrer Familie überliefert wurden.	○	○	○	○	○	○
Sie lässt keine Gelegenheit aus, Spaß zu haben. Es ist ihr wichtig, Dinge zu tun, die ihr Vergnügen bereiten.	○	○	○	○	○	○
Sie findet es wichtig, sich für Dinge zu interessieren. Sie ist gern neugierig und versucht gern, alle möglichen Sachen zu verstehen.	○	○	○	○	○	○
Es ist ihr wichtig, Menschen zu verzeihen, die ihr wehgetan haben. Sie versucht, das Gute in ihnen zu sehen und nicht nachtragend zu sein.	○	○	○	○	○	○
Es ist ihr wichtig, im Leben voranzukommen. Sie bemüht sich, besser zu sein als andere.	○	○	○	○	○	○
Sie glaubt, dass sie ihre Eltern und ältere Menschen immer mit Respekt behandeln sollte. Es ist ihr wichtig, gehorsam zu sein.	○	○	○	○	○	○

PV03_01 Es ist ihr wichtig, dass andere sie respektieren. Sie will, dass die Leute tun, was sie sagt.
PV03_02 Es ist ihr wichtig, ihren Freunden gegenüber loyal zu sein. Sie will sich für Menschen einsetzen, die ihr nahe stehen.
PV03_03 Sie ist fest davon überzeugt, dass die Menschen sich um die Natur kümmern sollten. Umweltschutz ist ihr wichtig.
PV03_04 Tradition ist ihr wichtig. Sie versucht, sich an die Sitten und Gebräuche zu halten, die ihr von ihrer Religion oder ihrer Familie überliefert wurden.
PV03_05 Sie lässt keine Gelegenheit aus, Spaß zu haben. Es ist ihr wichtig, Dinge zu tun, die ihr Vergnügen bereiten.
PV03_06 Sie findet es wichtig, sich für Dinge zu interessieren. Sie ist gern neugierig und versucht gern, alle möglichen Sachen zu verstehen.
PV03_07 Es ist ihr wichtig, Menschen zu verzeihen, die ihr wehgetan haben. Sie versucht, das Gute in ihnen zu sehen und nicht nachtragend zu sein.
PV03_08 Es ist ihr wichtig, im Leben voranzukommen. Sie bemüht sich, besser zu sein als andere.
PV03_09 Sie glaubt, dass sie ihre Eltern und ältere Menschen immer mit Respekt behandeln sollte. Es ist ihr wichtig, gehorsam zu sein.

1 = ist mir überhaupt nicht ähnlich
6 = ist mir sehr ähnlich
-9 = nicht beantwortet

PHP-Code

```
if (value('SD01') == 1) {
  goToPage('ende');
}
```

Seite 39

valmann

25. Im Folgenden beschreiben wir Ihnen einige Personen. Bitte lesen Sie jede Beschreibung und überlegen Sie, wie sehr Ihnen diese Person ähnelt oder nicht ähnelt.

Bitte kreuzen Sie an, wie ähnlich oder unähnlich Ihnen die jeweils beschriebene Person ist.

	ist mir überhaupt nicht ähnlich					ist mir sehr ähnlich
Es ist ihm wichtig, neue Ideen zu entwickeln und kreativ zu sein. Er macht Sachen gerne auf seine eigene originelle Art und Weise.	○	○	○	○	○	○
Es ist ihm wichtig, reich zu sein. Er möchte viel Geld haben und teure Sachen besitzen.	○	○	○	○	○	○
Er hält es für wichtig, dass alle Menschen auf der Welt gleich behandelt werden sollten. Er glaubt, dass jeder Mensch im Leben gleiche Chancen haben sollte.	○	○	○	○	○	○
Es ist ihm wichtig, seine Fähigkeiten zu zeigen. Er möchte, dass die Leute bewundern, was er tut.	○	○	○	○	○	○
Es ist ihm wichtig, in einem sicheren Umfeld zu leben. Er vermeidet alles, was seine Sicherheit gefährden könnte.	○	○	○	○	○	○
Er mag Überraschungen und hält immer Ausschau nach neuen Aktivitäten. Er denkt, dass im Leben Abwechslung wichtig ist.	○	○	○	○	○	○
Er glaubt, dass die Menschen tun sollten, was man Ihnen sagt. Er denkt, dass Menschen sich immer an Regeln halten sollten, selbst dann wenn es niemand sieht.	○	○	○	○	○	○
Es ist ihm wichtig, Menschen zuzuhören, die anders sind als er. Auch wenn er anderer Meinung ist als andere, will er die anderen trotzdem verstehen.	○	○	○	○	○	○

PV04_01 Es ist ihm wichtig, neue Ideen zu entwickeln und kreativ zu sein. Er macht Sachen gerne auf seine eigene originelle Art und Weise.
PV04_02 Es ist ihm wichtig, reich zu sein. Er möchte viel Geld haben und teure Sachen besitzen.
PV04_03 Er hält es für wichtig, dass alle Menschen auf der Welt gleich behandelt werden sollten. Er glaubt, dass jeder Mensch im Leben gleiche Chancen haben sollte.
PV04_04 Es ist ihm wichtig, seine Fähigkeiten zu zeigen. Er möchte, dass die Leute bewundern, was er tut.
PV04_05 Es ist ihm wichtig, in einem sicheren Umfeld zu leben. Er vermeidet alles, was seine Sicherheit gefährden könnte.
PV04_06 Er mag Überraschungen und hält immer Ausschau nach neuen Aktivitäten. Er denkt, dass im Leben Abwechslung wichtig ist.
PV04_07 Er glaubt, dass die Menschen tun sollten, was man Ihnen sagt. Er denkt, dass Menschen sich immer an Regeln halten sollten, selbst dann wenn es niemand sieht.
PV04_08 Es ist ihm wichtig, Menschen zuzuhören, die anders sind als er. Auch wenn er anderer Meinung ist als andere, will er die anderen trotzdem verstehen.

1 = ist mir überhaupt nicht ähnlich
6 = ist mir sehr ähnlich
-9 = nicht beantwortet

Seite 40

Bitte kreuzen Sie an, wie ähnlich oder unähnlich Ihnen die jeweils beschriebene Person ist.

	ist mir überhaupt nicht ähnlich					ist mir sehr ähnlich
Es ist ihm wichtig, zurückhaltend und bescheiden zu sein. Er versucht, die Aufmerksamkeit nicht auf sich zu lenken.	○	○	○	○	○	○
Es ist ihm wichtig, Spaß zu haben. Er gönnt sich selbst gerne etwas.	○	○	○	○	○	○

Es ist ihm wichtig, selbst zu entscheiden, was er tut. Er ist gerne frei und unabhängig von anderen.	○	○	○	○	○	○
Es ist ihm sehr wichtig, den Menschen um ihn herum zu helfen. Er will für deren Wohl sorgen.	○	○	○	○	○	○
Es ist ihm wichtig, sehr erfolgreich zu sein. Er hofft, dass die Leute seine Leistungen anerkennen.	○	○	○	○	○	○
Es ist ihm wichtig, dass der Staat seine persönliche Sicherheit vor allen Bedrohungen gewährleistet. Er will einen starken Staat, der seine Bürger verteidigt.	○	○	○	○	○	○
Er sucht das Abenteuer und geht gerne Risiken ein. Er will ein aufregendes Leben haben.	○	○	○	○	○	○
Es ist ihm wichtig, sich jederzeit korrekt zu verhalten. Er vermeidet es, Dinge zu tun, die andere Leute für falsch halten könnten.	○	○	○	○	○	○

PV05_01 Es ist ihm wichtig, zurückhaltend und bescheiden zu sein. Er versucht, die Aufmerksamkeit nicht auf sich zu lenken.
PV05_02 Es ist ihm wichtig, Spaß zu haben. Er gönnt sich selbst gerne etwas.
PV05_03 Es ist ihm wichtig, selbst zu entscheiden, was er tut. Er ist gerne frei und unabhängig von anderen.
PV05_04 Es ist ihm sehr wichtig, den Menschen um ihn herum zu helfen. Er will für deren Wohl sorgen.
PV05_05 Es ist ihm wichtig, sehr erfolgreich zu sein. Er hofft, dass die Leute seine Leistungen anerkennen.
PV05_06 Es ist ihm wichtig, dass der Staat seine persönliche Sicherheit vor allen Bedrohungen gewährleistet. Er will einen starken Staat, der seine Bürger verteidigt.
PV05_07 Er sucht das Abenteuer und geht gerne Risiken ein. Er will ein aufregendes Leben haben.
PV05_08 Es ist ihm wichtig, sich jederzeit korrekt zu verhalten. Er vermeidet es, Dinge zu tun, die andere Leute für falsch halten könnten.
1 = ist mir überhaupt nicht ähnlich
6 = ist mir sehr ähnlich
-9 = nicht beantwortet

Bitte kreuzen Sie an, wie ähnlich oder unähnlich Ihnen die jeweils beschriebene Person ist.

	ist mir überhaupt nicht ähnlich					ist mir sehr ähnlich
Es ist ihm wichtig, dass andere ihn respektieren. Er will, dass die Leute tun, was er sagt.	○	○	○	○	○	○
Es ist ihm wichtig, seinen Freunden gegenüber loyal zu sein. Er will sich für Menschen einsetzen, die ihm nahe stehen.	○	○	○	○	○	○
Er ist fest davon überzeugt, dass die Menschen sich um die Natur kümmern sollten. Umweltschutz ist ihm wichtig.	○	○	○	○	○	○
Tradition ist ihm wichtig. Er versucht, sich an die Sitten und Gebräuche zu halten, die ihm von seiner Religion oder seiner Familie überliefert wurden.	○	○	○	○	○	○
Er lässt keine Gelegenheit aus, Spaß zu haben. Es ist ihm wichtig, Dinge zu tun, die ihm Vergnügen bereiten.	○	○	○	○	○	○
Er findet es wichtig, sich für Dinge zu interessieren. Er ist gern neugierig und versucht gern, alle möglichen Sachen zu verstehen.	○	○	○	○	○	○
Es ist ihm wichtig, Menschen zu verzeihen, die ihm wehgetan haben. Er versucht, das Gute in ihnen zu sehen und nicht nachtragend zu sein.	○	○	○	○	○	○
Es ist ihm wichtig, im Leben voranzukommen. Er bemüht sich, besser zu sein als andere.	○	○	○	○	○	○
Er glaubt, dass er seine Eltern und ältere Menschen immer mit Respekt behandeln sollte. Es ist ihm wichtig, gehorsam zu sein.	○	○	○	○	○	○

PV06_01 Es ist ihm wichtig, dass andere ihn respektieren. Er will, dass die Leute tun, was er sagt.
PV06_02 Es ist ihm wichtig, seinen Freunden gegenüber loyal zu sein.

Er will sich für Menschen einsetzen, die ihm nahe stehen.
PV06_03 Er ist fest davon überzeugt, dass die Menschen sich um die Natur kümmern sollten. Umweltschutz ist ihm wichtig.
PV06_04 Tradition ist ihm wichtig. Er versucht, sich an die Sitten und Gebräuche zu halten, die ihm von seiner Religion oder seiner Familie überliefert wurden.
PV06_05 Er lässt keine Gelegenheit aus, Spaß zu haben. Es ist ihm wichtig, Dinge zu tun, die ihm Vergnügen bereiten.
PV06_06 Er findet es wichtig, sich für Dinge zu interessieren. Er ist gern neugierig und versucht gern, alle möglichen Sachen zu verstehen.
PV06_07 Es ist ihm wichtig, Menschen zu verzeihen, die ihm wehgetan haben. Er versucht, das Gute in ihnen zu sehen und nicht nachtragend zu sein.
PV06_08 Es ist ihm wichtig, im Leben voranzukommen. Er bemüht sich, besser zu sein als andere.
PV06_09 Er glaubt, dass er seine Eltern und ältere Menschen immer mit Respekt behandeln sollte. Es ist ihm wichtig, gehorsam zu sein.

1 = ist mir überhaupt nicht ähnlich
6 = ist mir sehr ähnlich
-9 = nicht beantwortet

26. Politisches Interesse

	überhaupt nicht					sehr stark
Wie stark interessieren Sie sich im Allgemeinen für Politik?	○	○	○	○	○	○

SD06_01 Wie stark interessieren Sie sich im Allgemeinen für Politik?
1 = überhaupt nicht
6 = sehr stark
-9 = nicht beantwortet

27. Bei politischen Einstellungen im Allgemeinen reden die Leute häufig von „links“ und „rechts“. Wo auf dieser Dimension würden Sie sich politisch verorten?

○	○	○	○	○	○	○	○	○	○	○
1 extrem links	2	3	4	5	6	7	8	9	10	11 extrem rechts

LR01 Links-Rechts Selbsteinschätzung
1 = 1 extrem links
2 = 2
3 = 3
4 = 4
5 = 5
6 = 6
7 = 7
8 = 8
9 = 9
10 = 10
11 = 11 extrem rechts
-9 = nicht beantwortet

28. In Deutschland neigen viele Leute längere Zeit einer bestimmten politischen Partei zu, obwohl sie auch ab und zu eine andere Partei wählen.

Wie ist das bei Ihnen: Neigen Sie – ganz allgemein – einer bestimmten Partei zu? Und wenn ja, welcher?

- ○ CDU
- ○ CSU
- ○ SPD
- ○ FDP
- ○ DIE LINKE
- ○ GRÜNE
- ○ PIRATEN
- ○ AfD
- ○ andere Partei, und zwar ________

- ○ Nein, neige keiner bestimmten Partei zu

SD07 PID
1 = CDU
2 = CSU
3 = SPD
4 = FDP
5 = DIE LINKE
6 = GRÜNE
7 = PIRATEN
8 = AfD
9 = andere Partei, und zwar
-1 = Nein, neige keiner bestimmten Partei zu
-9 = nicht beantwortet

SD07_09 andere Partei, und zwar
Offene Texteingabe

29. Wie stark oder wie schwach neigen Sie – alles zusammengenommen – dieser Partei zu?

○	○	○	○	○
sehr schwach	ziemlich schwach	mäßig	ziemlich stark	sehr stark

SD08 Stärke PID
1 = sehr schwach
2 = ziemlich schwach
3 = mäßig
4 = ziemlich stark
5 = sehr stark
-9 = nicht beantwortet

30. Sind Sie derzeitig berufstätig?

○ nicht berufstätig
○ teilzeit berufstätig
○ vollzeit berufstätig
○ Schüler/Student

SD05 Berufstätig?
1 = nicht berufstätig
2 = teilzeit berufstätig
3 = vollzeit berufstätig
4 = Schüler/Student
-9 = nicht beantwortet

Seite 43

PHP-Code

```
redirect('http://befragungen.keypanel.de/?m=6006&return=complete&securitykey=8TtpAFgfksEJexKMaxhZ&i_sur
```

Letzte Seite

Vielen Dank für Ihre Teilnahme!

Wir möchten uns ganz herzlich für Ihre Mithilfe bedanken.

Ihre Antworten wurden gespeichert, Sie können das Browser-Fenster nun schließen.

Verantwortliche:
Tobias Rothmund, Jun.-Prof. Dr.
Carsten Grimme, M. A.

Kontakt:
grimme@uni-landau.de
www.uni-koblenz-landau.de
Universität Koblenz-Landau, Campus Landau
Fortstraße 7
76829 Landau
